# Apolipoprotein E Isoform-Dependent Effects on Human Amyloid Precursor Protein/Aβ-Induced Behavioral Alterations and Cognitive Impairments and Insoluble Cortical Aβ42 Levels

**DOI:** 10.3389/fnagi.2022.767558

**Published:** 2022-03-01

**Authors:** Sarah Holden, Payel Kundu, Eileen R. S. Torres, Reetesh Sudhakar, Destine Krenik, Dmytro Grygoryev, Mitchel S. Turker, Jacob Raber

**Affiliations:** ^1^Department of Behavioral Neuroscience, Oregon Health & Science University, Portland, OR, United States; ^2^Oregon Institute of Occupational Health Sciences, Oregon Health & Science University, Portland, OR, United States; ^3^Department of Molecular and Medical Genetics, Oregon Health & Science University, Portland, OR, United States; ^4^Department of Neurology, Division of Neuroscience, Oregon National Primate Research Center, Oregon Health & Science University, Portland, OR, United States; ^5^Department of Psychiatry, Division of Neuroscience, Oregon National Primate Research Center, Oregon Health & Science University, Portland, OR, United States; ^6^Department of Radiation Medicine, Division of Neuroscience, Oregon National Primate Research Center, Oregon Health & Science University, Portland, OR, United States; ^7^College of Pharmacy, Oregon State University, Corvallis, OR, United States

**Keywords:** amyloid precursor protein, apolipoprotein E, genetic interactions, behavioral and cognitive performance, Aβ levels

## Abstract

Mice expressing human amyloid precursor protein (APP) containing the dominant Swedish and Iberian mutations (*App^NL–F^*) or also Arctic mutation (*App^NL–G–F^*) show neuropathology and hippocampus-dependent cognitive impairments pertinent to Alzheimer’s disease (AD) in mouse models at 18 and 6 months of age, respectively. Apolipoprotein E, involved in cholesterol metabolism, plays an important role in maintaining the brain. There are three human apolipoprotein E isoforms: E2, E3, and E4. Compared to E3, E4 increases while E2 protects against AD risk. At 6 months of age, prior to the onset of plaque pathology, E3, but not E4, protected against hAPP/Aβ-induced impairments in spatial memory retention in the Morris water maze. However, these earlier studies were limited as hapoE was not expressed outside the brain and E3 or E4 was not expressed under control of an apoE promotor, E2 was often not included, hAPP was transgenically overexpressed and both mouse and hAPP were present. Therefore, to determine whether apoE has isoform-dependent effects on hAPP/Aβ-induced behavioral alterations and cognitive impairments in adult female and male mice at 6 and 18 months of age, we crossed *App^NL–G–F^* and *App^NL–F^* mice with E2, E3, and E4 mice. To distinguish whether genotype differences seen at either time point were due to main effects of hAPP, hapoE, or hAPP × hapoE genetic interactions, we also behavioral and cognitively tested E2, E3, and E4 female and male mice at 6 and 18 months of age. We also compared behavioral and cognitive performance of 18-month-old *App^NL–G–F^* and *App^NL–F^* female and male mice on a murine apoE background along with that of age—and sex-matched C57BL/6J wild-type mice. For many behavioral measures at both time points there were APP × APOE interactions, supporting that apoE has isoform-dependent effects on hAPP/Aβ-induced behavioral and cognitive performance. NL-G-F/E3, but not NL-G-F/E2, mice had lower cortical insoluble Aβ42 levels than NL-G-F/E4 mice. NL-F/E3 and NL-F/E2 mice had lower cortical insoluble Aβ42 levels than NL-F/E4 mice. These results demonstrate that there are apoE isoform-dependent effects on hAPP/Aβ-induced behavioral alterations and cognitive impairments and cortical insoluble Aβ42 levels in mouse models containing only human APP and apoE.

## Introduction

The hippocampus, a brain area critical for learning and episodic memory, plays a key role in the progress of Alzheimer’s disease (AD) (Braak and Braak, 1991, 1998; Thal et al., 1999). The amyloid precursor protein (APP) has physiological functions in brain development, synaptic function, injury and neuroprotection (Muller et al., 2017). While oligomer aggregates of amyloid beta (Aβ) peptides generated from APP play a key role in AD neuropathology, physiological function of Aβ include modulation of synaptic function, memory consolidation (Karisetty et al., 2020). Dr. Saido at the Riken Institute generated human amyloid precursor protein (hAPP) knock-in (KI) mouse models expressing human APP at physiological levels (Saito et al., 2014, 2016). One of the models contains the Swedish and Iberian mutations (*App^NL–F^*) and shows neuropathology and hippocampus-dependent cognitive impairments at 18 months of age (Saito et al., 2014, 2016). Another model also includes a third Arctic mutation (*App^NL–G–F^*) and shows neuropathology, hippocampus-dependent cognitive impairments, and impaired long-term potentiation (LTP) in the hippocampus and cortex at 6 months of age (Saito et al., 2014, 2016; Latif-Herandez et al., 2016). Recently, we compared behavioral and cognitive performance in 6-month-old *App^NL–G–F^* and *App^NL–F^* mice which revealed that females were more affected than males (Kundu et al., 2021), consistent with the higher AD risk in women than men (Farrer et al., 1997; Mielke, 2018).

Apolipoprotein E is involved in cholesterol metabolism and plays an important role in development, maintenance, and repair of the brain following injury (Husain et al., 2021). There are three human apolipoprotein E isoforms: E2, E3, and E4. Compared to E3, E2 is protective with regard to AD risk (Raber et al., 2004; Spinney, 2014), more prevalent among centenarians, and associated with improved episodic memory performance, larger hippocampal volume, and reduced hippocampal atrophy (Chiang et al., 2010; Goldberg et al., 2020). Episodic memory is associated with the hippocampus and entorhinal cortex (Braak and Braak, 1991, 1998; Thal et al., 1999). Compared to E3, E4 is an AD risk factor, particularly in women (Farrer et al., 1997), and associated with accelerated hippocampal atrophy (Li et al., 2016). ApoE isoforms differ in binding to Aβ and E4 has been implicated in facilitating the transport of soluble Aβ to the synapse, eliciting neuropathology (Koffie et al., 2012).

We previously reported that at 6 months of age, prior to the onset of plaque pathology, E3, but not E4, was able to protect against hAPP/Aβ-induced impairments in spatial memory retention in the water maze (Raber et al., 2000). This and other earlier studies were limited as they involved crosses between human (h) apoE and hAPP transgenic mice in which hapoE was not expressed outside the brain and E3 or E4 was not expressed under control of an apoE promotor in the brain, E2 was often not included, hAPP was transgenically overexpressed and both mouse and hAPP were present (Holtzman et al., 1999; Raber et al., 2000; Hartman et al., 2001; Buttini et al., 2002; Fagan et al., 2002). A recent exception was a study that included hapoE TR but hAPP overexpressing mice showing a greater load of Aβ pathology in hAPP/E4 mice than APP/E2 mice and impairments in object recognition and radial arm maze performance in hAPP/E4 (Pankiewicz et al., 2014).

To determine whether apoE has isoform-dependent effects on hAPP/Aβ-induced behavioral alterations and cognitive impairments in adult female and male mice at 6 months of age, we crossed APP NL-G-F mice with E2, E3, and E4 mice to generate NL-G-F/E2, NL-G-F/E3, and NL-G-F/E4 mice. To determine whether apoE has isoform-dependent effects on hAPP/Aβ-induced behavioral alterations and cognitive impairments in aged female and male mice at 18 months of age, we crossed APP NL-F KI with E2, E3, and E4 mice to generate NL-F/E2, NL-F/E3, and NL-F/E4 mice. To distinguish whether genotype differences seen at either time point were due to main effects of hAPP, hapoE, or hAPP × hapoE genetic interactions, we also behaviorally and cognitively tested E2, E3, and E4 female and male mice at 6 and 18 months of age. Separately, we compared behavioral and cognitive performance of 18-month-old *App^NL–G–F^* and *App^NL–F^* female and male mice on a murine apoE background along with that of age-and sex-matched C57BL/6J wild-type mice. In addition, in a subset of hAPP mice we analyzed the cortical Aβ levels.

## Materials and Methods

### Animals

We started to cross hAPP knock-in (KI) mice containing the Swedish and Iberian mutations (*App^NL–F^*) or the Arctic mutation included as third mutation (*App^NL–G–F^*) generated by Dr. Saido (Saito et al., 2014, 2016) on a C57BL/6J background with human E2, E3, and E4 mice generated by Dr. Sullivan (Sullivan et al., 1997, 1998; Knouff et al., 1999) and commonly known as knockin or targeted replacement mice since the human coding region replaces the mouse apoE coding region, on 12/27/17. The offspring were genotyped and the bigenic mice heterozygous for hAPP and the human apoE isoforms crossed with each other to generate NL-G-F/E2, NL-G-F/E3, NL-G-F/E4, NL-F/E2, NL-F/E3, and NL-F/E4 mice. The APP knockin (KI) genotyping protocols are available on the Riken Institute web site (Saido and Al, 2014; Saido, 2016). Briefly, for genotyping of the Artic mutation, we used the following primers to amplify part of exon 17 containing the Artic mutation: Left primer: TGCTCATTGTTCCAGAGACG; Right primer: GTGATGACAATCACGGTTGC; product size: 238 kb; annealing temperature (Ta) 50°C. The PCR products were digested with the MboII restriction enzyme:


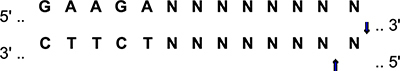


The digestion of WT APP produces 171 bp and 67 bp fragments. The presence of the Arctic mutation prevents digestion:


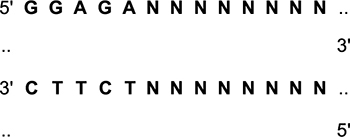


For genotyping of the Iberian mutations, we used the following primers to amplify part of exon 17 containing the Iberian mutation: Left primer: CCTTTTTCTCGGCTTCCTTT; right primer: CACTTGCAGACAAGCCTCCA; product size: 203 kb; annealing temperature: (Ta) 50°C. The PCR products were digested with the BsaBI restriction enzyme:


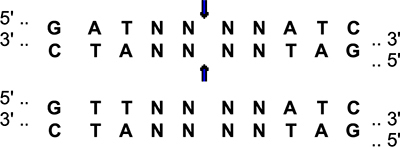


Digestion of WT APP produces 110 bp and 93 bp fragments. The presence of the Iberian mutation prevents digestion. ApoE genotyping was performed as described (Zhong et al., 2016).

Homozygous matings were used to generate the mice for the proposed experiments. In addition, we used E2, E3, and E4 mice for behavioral and cognitive testing at the two time points. We also included 18-month-old APP KI mice on a murine apoE background. The behavioral and cognitive performance of 6-month-old *App^NL–G–F^* and *App^NL–F^* female and male mice on a murine apoE background along with that of age—and sex-matched C57BL/6J wild-type mice we reported in an earlier study (Kundu et al., 2021).

The breeding success for generating the required number of mice of the desired genotypes and the enrollment of mice for the 6- and 18-month time points are indicated in Table 1. We also included C57BL/6J wild-type (WT) mice as an additional control group for the 18-month time point but for clarity did not include their breeder information in Table 1.

**TABLE 1:** Breeding success for generating the mice with the desired genotypes and experimental mice for the 6- and 18-month time points^1^.

Genotype	BP	Litters	Offspring	Average litter size	*F*	*M*
***6-month time point***						
E2	3	15	86	5.73	12	12
E3	5	17	108	6.35	14	14
E4	7	36	235	6.53	14	15
NL-G-F/E2	3	15	124	8.27	5	7
NL-G-F/E3	2	8	38	4.75	13	8
NL-G-F/E4	1	5	38	7.6	10	13
NL-F	4	15	87	5.8	12	15
NL-G-F	5	13	78	6	16	17
***18-month time point***						
E2	8	33	208	6.30	17	17
E3	6	29	179	6.17	7	13
E4	5	25	162	6.48	8	15
NL-F/E2	2	6	48	8	9	9
NL-F/E3	1	4	26	6.5	9	9
NL-F/E4	2	9	55	6.11	5	7
NL-F	4	15	87	5.8	12	10
NL-G-F	4	11	68	6.18	11	11

*^1^BP, number of breeding pairs; litters, number of litter; offspring, number of offspring; F, females; M, males.*

*BP, litters, offspring, and average litter size are important measures to consider, especially in studies involving mouse models containing dominant AD genetic mutations combined with AD genetic risk factor that might affect fertility and litter size.*

Mice were tested at approximately 6 or 18 months of age. Mice were group housed in standard vivarium conditions until the start of the study. The animals were then singly housed for circadian home cage activity monitoring, as described below, and remained singly housed for the duration of the experiment. The vivarium was maintained at 20–21°C and food (PicoLab Rodent Diet 20, no. 5053; PMI Nutrition International, St. Louis, MO, United States) was available *ad libitum.* Lights were kept on a 12 h light:12 h dark cycle. Testing and data analyses were conducted by experimenters blinded to the genotype of the mice. Procedures complied with the NIH Guide for the Care and Use of Laboratory Animals and with IACUC approval at Oregon Health and Science University (OHSU) and were consistent with the ARRIVE guidelines.

### Behavioral and Cognitive Assessment Group Sizes and Test Order

Behavioral and cognitive tests were conducted in the order illustrated in Table 2: circadian home cage activity, quality of nest building, measures of anxiety in the elevated zero maze, performance on the wire hang test, measures of activity and anxiety in the open field, object recognition, spontaneous alternation in the Y maze, and contextual fear learning and memory. This order was selected to start with the anticipated least stress-inducing test and end with the anticipated most stress-inducing test. The mazes were surrounded with a white curtain in order to isolate the mice from the surrounding room and the experimenter.

**TABLE 2:** Overview of behavioral and cognitive testing.

*Week*	*Test*	*Measures*
1	Home cage activity	Circadian activity
1	Nest building	Quality of nest building
2 (days 1–2)	Elevated zero maze	Measures of activity and anxiety
2 (days 4–5)	Wire hang test	Muscle function and coordination
3 (days 1–3)	Open field	Measures of activity and anxiety
3 (days 4–5)	Object recognition	Learning and memory (cognition)
4 (day 1)	Y maze	Spontaneous alternation (cognition)
4 (days 2–4)	Contextual and cued fear learning and memory	Fear learning and memory

#### Circadian Home Cage Activity Monitoring

Home cage activity was monitored using infrared home-cage activity sensors (Biobserve, St. Augustin, Germany), as previously described (Johnson et al., 2016). Mice were individually housed during activity monitoring. Activity counts were measured and data were expressed as mean activity count per hour. In addition to analysis of the activity during the light and dark periods, the ratio of circadian activity, defined as activity during the dark period divided by that of the light period was analyzed as well.

#### Nest Building

Nest building was measured at two time points, once after the conclusion of activity monitoring and again after the conclusion of novel object recognition testing. The protocol described in Deacon (2006) was used for scoring the quality of the nests (Johnson et al., 2015). Mice were placed into a clean cage with 3 g of pressed cotton square nestlet. After 24 h, nests were photographed for later scoring on a five-point nest rating scale. A score of 5 indicates the most complete and well-structured nest, whereas a score of 1 indicates the least functional nest. Scores were assigned in 0.5 point increments.

#### Elevated Zero Maze

Measures of anxiety were assessed in the elevated zero maze as previously described (Siegel et al., 2012). The elevated zero maze consisted of two open and two closed areas (each 35.5 cm in length; Hamilton-Kinder, Poway, CA, United States). The closed areas were surrounded by opaque walls (15 cm tall). Mice were placed into the maze into one of the open areas and allowed to explore for a single 10-min trial. Distance moved and the percentage of time spent in the open areas were analyzed with Motor Monitor software (Kinder Scientific, Poway, CA, United States). The zero maze was cleaned with 0.5% acetic acid between trials.

#### Wire Hang Test

A combination of motor function, balance, endurance, and muscle strength was assessed using the wire hang task, adopting the “falls and reaches” method described by van Putten (2011). Mice were placed on a 2 mm metal wire suspended between two metal posts, 35 cm above soft bedding so that they were hanging only by their front paws. Initial placement onto the wire was with the forepaws only. Once the trial began, use of back paws was also allowed. Mice started with a “fall score” of 10 and a “reach score” of 0. Over the duration of 180 s, one point was deducted from the fall score each time the animal fell, and one point was added each time the animal reached to one of the poles on either end of the wire. The time of each fall or reach event was also recorded. At the instance of either event, the timer was paused to place the mouse again on the center of the wire.

#### Y-Maze

Activity levels and hippocampus-dependent spontaneous alternations were assessed in the Y-maze. The Y-shaped maze (O’ Hara & Co., Ltd, Tokyo, Japan) has raised sides (3.8 cm bottom width, 12.55 cm top width, 12.55 cm height) with plastic, opaque gray arms (37.98 cm length) at a 120° angle from each other. This vendor was selected as the same Y-maze was used in the original study of the *App^NL–G–F^* and *App^NL–F^* mice (Saito et al., 2014) and our previous study in 6-month-old *App^NL–G–F^* and *App^NL–F^* mice (Kundu et al., 2021). Mice were placed in the center of the maze at the beginning of a 5-min trial. The maze was cleaned with 0.5% acetic acid between trials. Performance of the mice was recorded using Ethovision 15 XT software, Noldus Information Technology, Wageningen, Netherlands. Digital videos were later analyzed using hand scoring to measure the number of arm entries and to calculate the percent spontaneous alternations, a cognitive performance measure based on the innate tendency of rodents to explore a prior unexplored arm of the Y-maze. The criteria for an arm entry was when all four limbs were within the arm. The spontaneous alternation percentage was calculated by dividing the number of 3-arm alternations by the number of possible 3-arm alternations and multiplying the value by 100.

#### Performance in the Open Field and Object Recognition

Exploratory activity and measures of anxiety were assessed in the open field test. The open field consisted of a well-lit square (L 40.6 × W 40.6 × H 40.6 cm) with a central light intensity of 100 lux. Mice were allowed to explore the open field for 5 min during three consecutive days. On day 4, mice were exposed to the open field containing two identical objects for a 15-min trial. The objects were placed 10 cm apart and 15 cm from the adjacent walls of the arena. On day 5, one object was replaced with a novel object and mice were allowed to explore the open field for 15 min. Object exploration was measured manually as time when the mouse’s nose point was within 2 cm of the object. The enclosures were cleaned with 0.5% acetic acid between trials. Performance of mice was tracked using Ethovision 15 XT software. Time spent in the center of the open field was analyzed to assess measures of anxiety. Percent time spent with the novel object was calculated as the number of seconds spent exploring the novel object divided by the total number of seconds spent exploring the novel and familiar objects combined.

#### Contextual and Cued Fear Conditioning

Fear conditioning was assessed over the course of three consecutive days using a Med Associates mouse fear conditioning system (PMED-VFC-NIR-M, Med Associates, St. Albans, VT, United States) and Med Associates VideoFreeze automated scoring system. Mice were placed inside the fear conditioning chamber, where chamber lights were turned on at the beginning of the trial. The first day of testing consisted of a 5-min habituation phase during which no tone or shock was administered. Day two consisted of the acquisition phase, which lasted for a total of 5 min per trial. Following a 120-s baseline habituation period, two 30-s tones (80 dB) were presented, separated by 60-s inter-stimulus-intervals (ISIs). There was also a 60-s interval after the second tone-shock period. During the last 2 s of each tone, a 0.30 mA foot shock was administered. Twenty-four hours later the hippocampus-dependent contextual fear memory was assessed by placing the animals into the same chamber as in the acquisition phase for a 5-min trial. The chamber lights were on, but no tones or shocks were presented. Between habituation, acquisition and context trials, the chambers were thoroughly cleaned with 0.5% acetic acid.

Two hours following the contextual fear memory test, hippocampus-independent cued fear memory was assessed. Mice were placed in a novel environment, containing a novel floor texture, angled walls and vanilla extract soaked nestlet fixed to the outside of the wall. Each cued memory trial lasted a total of 6 min, consisting of a 180-s baseline period followed by a 180-s period during which the same tone used during training was presented. No shocks were administered during this phase of testing. Between cued trials, the chambers were thoroughly cleaned with 10% isopropanol.

#### Cortical Aβ Enzyme-Linked Immunosorbent Assay

Cortices of a subset of 6-month-old, NL-G-F/E2, NL-G-F/E3, NL-G-F/E4, NL-G-F, and NL-F and 18-month-old NL-F/E2, NL-F/E3, NL-F/E4, NL-G-F, and NL-F mice (*n* = 4 samples/sex/genotype/time point) were processed for analyses of soluble and insoluble Aβ40 and Aβ42 levels using Invitrogen ELISA kits (catalog numbers KHB3481 and KHB3441, respectively; Thermo Fisher Scientific, Waltham, MA, United States), according to the recommended guidelines in the production information sheets. To a thawed cortical tissue sample (both hemispheres), 400 μl of buffer A (phosphate-buffered saline containing a protease inhibitor tablet (cOmplete™, 11836170001 Roche, Millipore Sigma, Burlington, MA and filtered before use) was added. The tissue was homogenized using a Polytron for 10 s and subsequent a sonicator, and centrifuged at 45,000 rpm for 20 min at 4°C. The supernatant was collected as the soluble fraction. The same volume of buffer A was used to loosen the pellet. The sample was centrifuged again at 45,000 rpm for 5 min at 4°C. After removing the supernatant in a separate tube. the pellet was dissolved in Buffer B (containing 6 M Guanidine H-Cl and 50 mM Tris and filtered before use) and incubated at room temperature for 1 h. After this incubation, the sample was sonicated for 20 s and the extracted pellet was centrifuged at 45,000 rpm for 20 min at 4°C. The supernatant was collected as the insoluble fraction. Pilot experiments were performed to determine the optimal sample dilution. For the analyses of insoluble Aβ levels in 6-month-old mice, the tissue samples were diluted 1:4,000. For analysis of the soluble Aβ levels in 6-month-old mice, undiluted tissues samples were used. Standard curves were generated with the same buffer dilution as the samples. For the analyses of insoluble Aβ levels in 18-month-old mice, the tissue samples were diluted 1:20,000. For analysis of the soluble Aβ levels in 18-month-old mice, tissues samples were diluted 1:50. The ELISAs were read at 450 nm using a SpectraMax iD5 Multi-Mode Microplate Reader (Molecular Devices, VWR 76175-474, San Jose, CA, United States). The standard curves were generated and the levels in the samples determine using GraphPad Prism software, San Diego, CA, United States). Total protein amounts in the samples were determined by BCA protein assay kit (Pierce, Thermo Scientific, catalog #23225, Waltham, MA, United States) and reading the samples at 562 nm using the iD5 Reader.

### Statistical Analysis

Behavioral and cognitive data are expressed as mean ± SEM. Performance measures for each behavioral and cognitive test were analyzed using ANOVA or repeated-measures ANOVA. In the case of repeated measures ANOVA, sphericity was tested and Greenhouse–Geisser corrections were used when appropriate. Statistical significance was determined using an error probability level of *p* < 0.05. As we expected sex-dependent genotype effects, the behavioral and cognitive data were analyzed separately in females and males.

To determine whether apoE has isoform-dependent effects on hAPP/Aβ-induced behavioral alterations and cognitive impairments in adult female and male mice at 6 months of age, we analyzed the behavioral and cognitive performance of the following six genotypes: NL-G-F/E2, NL-G-F/E3, NL-G-F/E4, E2, E3, and E4 mice. To determine whether apoE has isoform-dependent effects on hAPP/Aβ-induced behavioral alterations and cognitive impairments in aged female and male mice at 18 months of age, we analyzed the behavioral and cognitive performance of the following six genotypes: NL-F/E2, NL-F/E3, NL-F/E4, E2, E3, and E4 mice. We separately analyzed the behavioral and cognitive performance of 18-month-old *App^NL–F^*, *App^NL–G–F^*, and WT female and male mice on the murine apoE background.

Data were analyzed using SPSS Statistics for Windows (Version 25, Armonk, NY, United States; IBM Corp., Chicago, IL, United States). All figures were generated using GraphPad Prism software (version 8, San Diego, CA, United States).

## Results

### Behavioral and Cognitive Performance of E2, E3, E4, NL-G-F/E2, NL-G-F/E3, and NL-G-F/E4 Mice at the 6-Month Time Point

The results of the statistical analyses of the behavioral and cognitive performance at the 6-month time point are summarized in Supplementary Table 1A.

#### Body Weights

**FIGURE 1:**
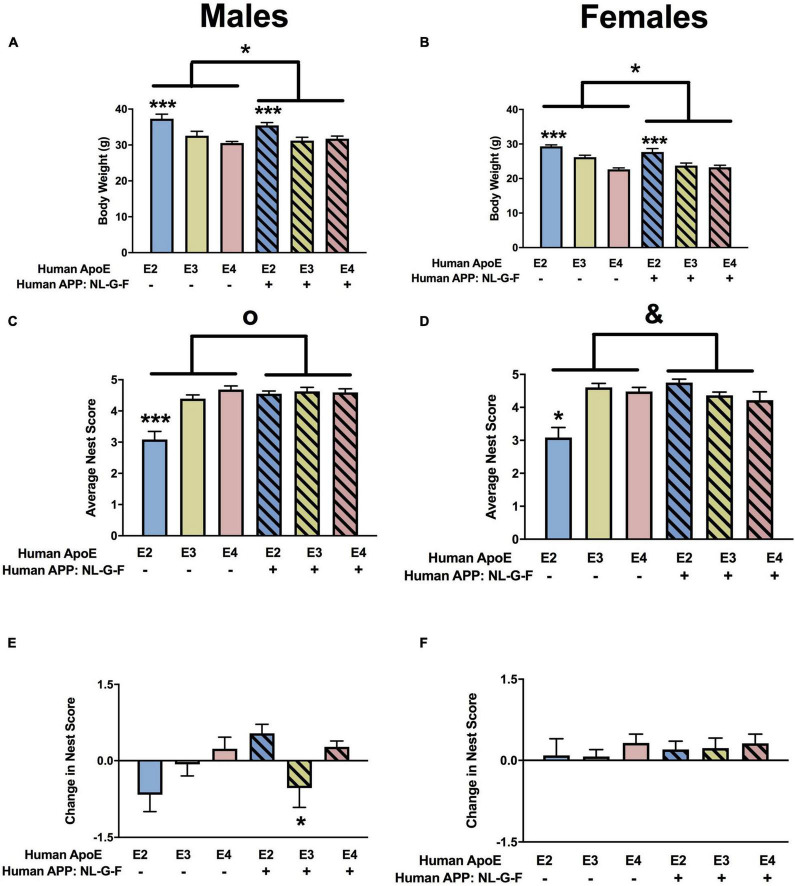
Body weights and nest score measures in males **(A,C,E)** and females **(B,D,F)** at the 6-month time point. **(A)** There was an effect of APOE and APP on body weights in males. E2 males were heavier than E3 and E4 males. ***p < 0.001. Mice were lighter in the presence of NL-G-F (mean body weights males; mAPP: 32.65 g; NL-G-F: 31.6 g; mean body weights females; mAPP: 25.8 g; NL-G-F: 24.4 g). *p < 0.05. **(B)** There was an effect of APOE and APP on body weights and an APP × APOE interaction in females. E2 males were heavier than E3 and E4 females. ***p < 0.001. Mice were lighter in the presence of NL-G-F. *p < 0.05. **(C)** There was an effect of APOE and APP and an APP × APOE interaction for nest scores in males. Nest score were lower in E2 than E3 and E4 males. ***p < 0.001. Nest score were higher in the presence of NL-G-F. *^o^p* < 0.001. This was most profound in the E2 background. **(D)** There was an effect of APOE and APP and an APP × APOE interaction for nest scores in females. Nest score were lower in E2 than E3 and E4 females. *p < 0.05. Nest score were higher in the presence of NL-G-F. *^o^p* < 0.001. This was most profound in the E2 background. ^&^*p* = 0.013. **(E)** There was an APP × APOE interaction for the change in nest scores in males. The change in nest score was worse in NL-G-F/E3 than NL-G-F/E2 or NL-G-F/E4 mice. *p < 0.05. **(F)** There were no effects on the change in nest scores in females.

For body weights, there were effects of APOE (Figure 1A) and APP (Figure 1B) in males and females. Body weights were higher in E2 than sex-matched E3 or E4 mice and body weights were higher in apoE mice than sex-matched NL-G-F/apoE mice. In addition, in females there was an APP × APOE interaction. While body weights were higher in NL-G-F/E2 than E2 female mice, this effect was not seen in the E3 or E4 female mice (Figure 1B).

#### Nest Building

For quality of nest building, there were effects of APOE, APP, and an APP × APOE interaction in males (Figure 1C) and females (Figure 1D). In males and females, nest building scores were lower in E2 than E3 or E4 mice. While body weights were higher in NL-G-F/E2 than E2 mice, this effect was not seen in the E3 or E4 background. The quality of nest building was analyzed at two time points, once after the conclusion of activity monitoring and again after the conclusion of novel object recognition testing. When the change in nest score was analyzed, there was an APP × APOE interaction and a trend toward an effect of APOE in males (Figure 1E). While E2 males showed a negative change score, indicating a worsened nest score over time, NL-G-F/E2 mice showed a positive change score, indicating an improved nest score over time. In contrast, in E3 mice, change scores were worse in NL-G-F/E3 than E3 mice. Change scores were comparable in E4 and NL-G-F/E4 mice. There were no effects on change scores and no negative change scores in females (Figure 1F).

#### Home Cage Activity

**FIGURE 2:**
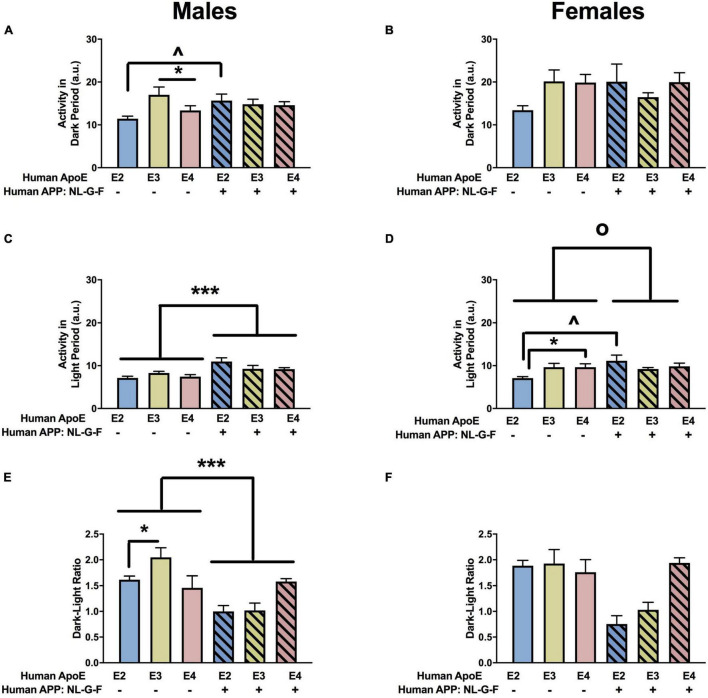
Home cage activity in males **(A,C,E)** and females **(B,D,F)** at the 6-month time point. **(A)** E3 males were more active than E4 males during the dark period. *p < 0.05. In addition, NL-G-F/E2 males were more active than E2 males during the dark period. ^∧^*p* < 0.05. **(B)** Activity levels of females during the dark period. **(C)** There was an effect of APP on activity during the light period in males. ***p < 0.001. **(D)** There was an effect of APP and an APP × APOE interaction for activity during the light in females. *^o^p* = 0.043. E2 females were less active than E4 females during the light period. *p < 0.05. Activity levels during the light period were higher in NL-G-F/E2 than E2 females. ^∧^*p* < 0.05. **(E)** There was an effect of APP and APOE on the dark/light ratio in males. Dark/light ratios were lower in NL-G-F males. ***p < 0.001. In addition, the dark/light ratio was higher in E3 than E2 males. *p < 0.05. **(F)** Dark/light ratio of females during home cage activity analysis.

When circadian activity levels were assessed in the home cage, there were trends toward APP × APOE interaction for activity during the dark period in males (Figure 2A) and females (Figure 2B) but they did not reach significance. However, there was an effect of APP on activity during the light period in males (Figure 2C) and females (Figure 2D). In females, there was also an APP × APOE interaction for activity during the light period (Figure 2D). In males, activity levels during the light period were higher in NL-G-F/E2, NL-G-F/E3, and NL-G-F/E4 than E2, E3, and E4 mice. In contrast, in females, activity levels during the light period were only higher in NL-G-F/E2 than E2 mice. Similar to what was seen for the light period, there was an effect of APP on the ratio dark/light activity in males (Figure 2E), with lower dark/light ratios in NL-G-F/E2, NL-G-F/E3, and NL-G-F/E4 than E2, E3, and E4 mice. This effect was not seen in females (Figure 2F). In addition, in males, the dark/light ratio was higher in E3 than E2 mice. The effects seen during the light period and the dark/light ratio are consistent with a detrimental effect of APP on activity levels during the period the mice are normally resting.

#### Elevated Zero Maze

**FIGURE 3:**
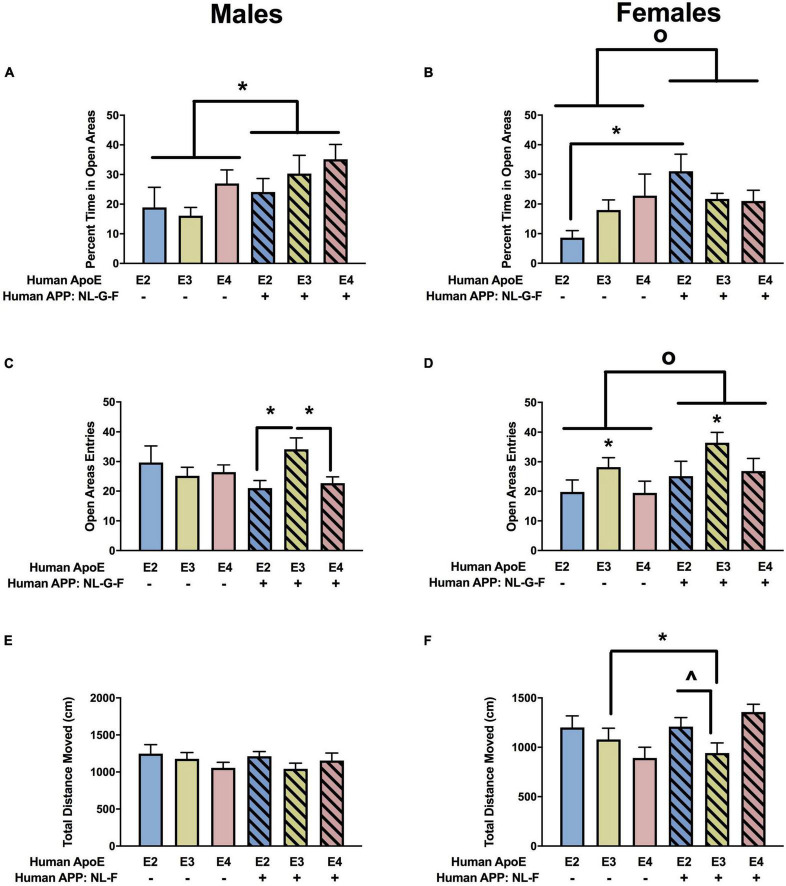
Performance of males **(A,C,E)** and females **(B,D,F)** in the elevated zero maze at the 6-month time point. **(A)** There was an effect of APP on measures of anxiety in males. Measures of anxiety were lower in NL-G-F mice. *p < 0.05. **(B)** There was an effect of APP and an APP × APOE interaction for measures of anxiety in females. Measures of anxiety were lower in NL-G-F mice. *^o^p* = 0.023. In addition, measures of anxiety were lower in NL-G-F/E2 than E2 females. *p < 0.05. **(C)** There was an APP × APOE interaction for entries into the open areas in males. NL-G-F/E3 males entered the open areas more than NL-G-F/E2 and NL-G-F/E4 males. *p < 0.05. **(D)** There was an effect of APP and APOE on entries into the open areas in females. NL-G-F females entered the open areas more often. *^o^p* = 0.039. In addition, measures of anxiety were lower in E3 than E2 and E4 females. *p < 0.05. **(E)** Activity levels of males in the elevated zero maze. **(F)** There was an APP × APOE interaction for activity levels of females in the elevated zero maze. E3 females were more active than NL-G-F/E3 females. *p < 0.05. In addition, NL-G-F/E3 females were less active than NL-G-F/E2 and NL-G-F/E4 females. ^∧^*p* < 0.05.

Next, measures of anxiety were assessed in the elevated zero maze. There was an effect of APP on the percent time spent in the more anxiety-provoking open areas in males (Figure 3A) and females (Figure 3B). In females, there was also an APP × APOE interaction for the percent time spent in the open areas (Figure 3B). In males, the percent time spent in the open areas was lower in NL-G-F/E2, NL-G-F/E3, and NL-G-F/E4 than E2, E3, and E4 mice. In contrast, in females, the percent time spent in the open areas was only higher in NL-G-F/E2 than E2 mice.

When entries into the open areas were analyzed, there was an APP × APOE interaction in the males (Figure 3C) and effects of APOE and APP in the females (Figure 3D). In males, open arm entries were higher in NL-G-F/E3 than NL-G-F/E2 or NL-G-F/E4 mice. This pattern was not seen in E2, E3, and E4 male mice. There were no effects of APOE or APP on activity levels of the males in the elevated zero maze (Figure 3E). However, there was an APP x APOE interaction for activity levels of females in the elevated zero maze (Figure 3F).

#### Wire Hang

**FIGURE 4:**
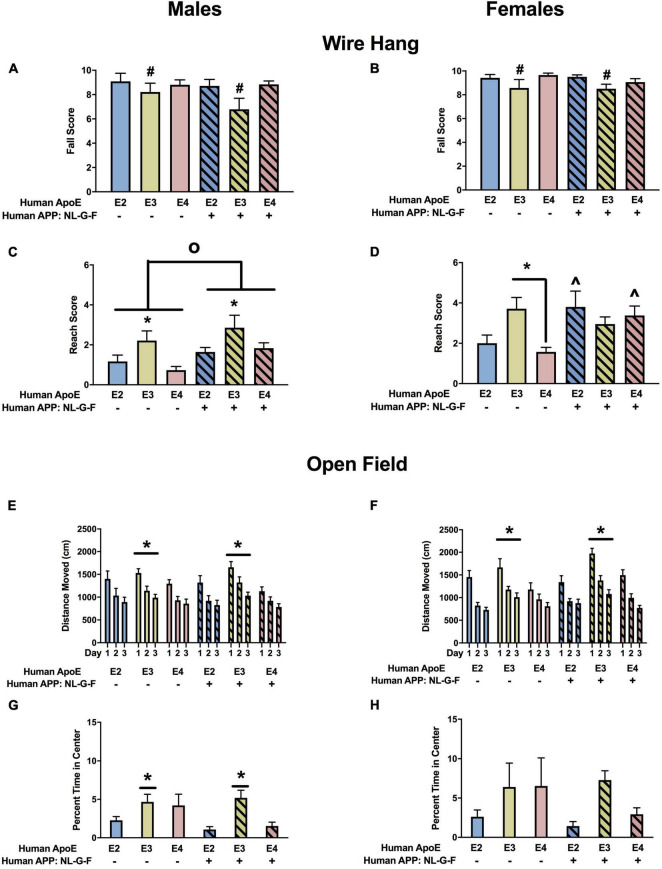
Performance of males **(A,C,E,G)** and females **(B,D,F,H)** in the wire hang and open field tests at the 6-month time point. **(A)** There was an effect of APOE on fall scores of males in the wire hang test. There was a trend toward lower fall scores in E3 than E2 (*p* = 0.065) and E4 males (*p* = 0.050). **(B)** There also was an effect of APOE on fall scores of females in the wire hang test. There was a trend toward lower fall scores in E3 than E2 females (*p* = 0.076). **(C)** There was an effect of APOE and APP on reach scores of males in the wire hang test. Reach score were higher in E3 than E2 and E4 males. *p < 0.05. In addition, reach scores were higher in NL-G-F mice. *^o^p* < 0.05. **(D)** There was an effect of APP and an APP × APOE interaction for reach scores of females in the wire hang test. Reach scores were higher in E3 than E4 females. *p < 0.05. Reach score were higher in NL-G-F/E2 and NL-G-F/E4 than E2 and E4 females, respectively. ^∧^*p* < 0.05. **(E)** There was an effect of APOE on activity levels of males in the open field test. E3 males were more active than E4 males. *p < 0.05. **(F)** There also was an effect of APOE on activity levels of females in the open field test. E3 females were more active than E2 and E4 females. *p < 0.05. **(G)** In males, NL-G-F/E2 and NL-G-F/E4 mice spent less time in the center of the open field than E2 and E4 mice, respectively. This was apoE isoform-dependent and not seen in NL-G-F/E3 and E3 mice. In the absence of hAPP, E2 mice spent less time in the center of the open field than E3 or E4 mice. In contrast, in the presence of hAPP, NL-G-F/E2 and NL-G-F/E4 mice spent less time in the center of the open field than NL-G-F/E3 mice. *p < 0.05. **(H)** In females, E2 mice spent less time in the center of the open field than E3 or E4 mice. *p < 0.05.

There was an effect of APOE on fall scores in males (Figure 4A) and females (Figure 4B). In the absence and presence of hAPP, there was a trend toward lower fall scores in E3 than E2 and E4 males and a trend toward lower fall scores in E3 than E2 females. In males, there were effects of APOE and APP on reach scores (Figure 4C). In the absence and presence of hAPP, reach scores were higher in E3 than E2 or E4 mice. In addition, reach scores were higher in NL-G-F/E2, NL-G-F/E3, and NL-G-F/E4 mice than apoE genotype-matched mice. In females, there was an effect of APP on reach scores and an APP × APOE interaction (Figure 4D). Reach scores were higher in NL-G-F/E2 and NL-G-F/E4 mice than E2 and E4 mice. In addition, reach scores were higher in E3 than E2 or E4 mice in the absence but not in the presence of hAPP.

#### Open Field

When activity levels were analyzed during three subsequent days of open field testing, there was an effect of APOE in males (Figure 4E) and females (Figure 4F). In males, activity levels were higher in E3 than E4 mice, and in females activity levels were higher in E3 than E2 or E4 mice.

The time spent in the more anxiety-provoking center area of the open field was assessed as well. In males, there were effects of APOE and APP and an APP × APOE interaction (Figure 4G). NL-G-F/E2 and NL-G-F/E4 mice spent less time in the center of the open field than E2 and E4 mice, respectively. This was apoE isoform-dependent and not seen in NL-G-F/E3 and E3 mice. In the absence of hAPP, E2 mice spent less time in the center of the open field than E3 or E4 mice. In contrast, in the presence of hAPP, NL-G-F/E2 and NL-G-F/E4 mice spent less time in the center of the open field than NL-G-F/E3 mice. In females, E2 mice spent less time in the center of the open field than E3 or E4 mice (Figure 4H).

#### Y Maze

When spontaneous alternation was analyzed in the Y maze, there were no effects of APP, APOE, or APP × APOE interactions in males (Supplementary Figure 1A) or females (Supplementary Figure 1B). When activity levels were analyzed in the Y maze, there were effects of APOE and APP in males (Supplementary Figure 1C) and females (Supplementary Figure 1D). In males, activity levels were higher in E2 than E3 or E4 mice. In females, activity levels were higher in E2 than in E4 mice. In addition activity levels were lower in the presence of hAPP NL-G-F than in its absence in males (Supplementary Figure 1C) and females (Supplementary Figure 1D).

#### Fear Conditioning

**FIGURE 5:**
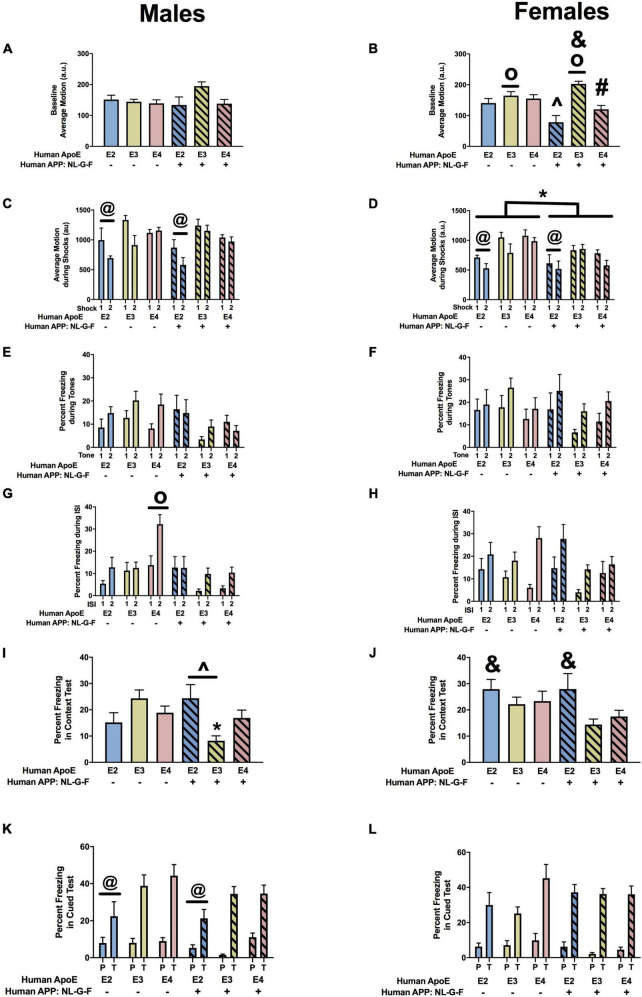
Performance of males **(A,C,E,G,I,K)** and females **(B,D,F,H,J,L)** in the fear conditioning test at the 6-month time point. **(A)** Baseline motion of males prior to the first tone-shock pairing. **(B)** There was an effect of APOE, an APP × APOE interaction, and a trend toward an effect of APP for baseline motion of females. Baseline motion was higher in E3 than E2 and E4 females. *^o^p* < 0.05. In addition, baseline motion was higher in NL-G-F/E3 than NL-G-F/E2 and NL-G-F/E4 females. ^&^*p* < 0.05. In addition, NL-G-F/E2 females showed lower baseline activity than E2 females. ^∧^*p* < 0.05. In contrast NL-G-F/E3 females had higher baseline activity levels than E3 females. ^&^*p* < 0.05. There was a trend toward a lower baseline activity in NL-G-F/E4 than E4 females (*p* = 0.066). **(C)** There was an effect of APOE on motion during the shocks of males. E2 males responded less in response to the shocks than E3 and E4 males. ^@^*p* < 0.05. **(D)** There was an effect of APOE on motion during the shocks of females. E2 females responded less in response to the shocks than E3 and E4 females. ^@^*p* < 0.05. In addition, NL-G-F females responded less to the shocks. *p < 0.05. **(E)** There was an effect of APP on freezing during the tones. While NL-G-F mice froze more during the first tone, they froze less during the second tone. **(F)** Freezing during the tones of females. **(G)** There was an effect of APOE, APP, and an APP × APOE interaction for freezing of males during the ISIs. E4 males froze more during the ISIs than NL-G-F/E4 males. *^o^p* < 0.05. **(H)** There was an APP × APOE interaction for freezing of females during the ISIs. While NL-G-F/E4 females froze more than E4 females during the first ISI, they froze less than E4 females during the second ISI. **(I)** There was an APP × APOE interaction for freezing of males during the contextual memory test. NL-G-F/E3 males froze less than E3 males in the contextual memory test. *p < 0.05. In addition, NL-G-F-E2 males froze more than NL-G-F/E3 males in the contextual memory test. **(J)** There was an effect of APOE on contextual fear memory in females. E2 females froze more than E3 females in the contextual memory test. ^&^*p* < 0.05. **(K)** There was an effect of APOE on cued fear memory in males. E2 males froze less than E3 and E4 males in the cued fear memory test. ^@^*p* < 0.05. **(L)** Cued fear memory of females.

When we analyzed the activity levels prior to the first tone during fear learning (baseline motion), there were no effects in males (Figure 5A). However, in females, there was an effect of APOE, an APP × APOE interaction, and a trend toward an effect of APP (Figure 5B). Baseline motion was lower in E2 than E3 or E4 females. In addition, in the presence of hAPP, baselines motion was higher in NL-G-F/E3 than NL-G-F/E2 and NL-G-F/E4 females. When the response to the shocks was analyzed, there was an effect of APOE in males (Figure 5C) and females (Figure 5D). In addition, there was an effect of APP in females (Figure 5D). In males and females, the response to the shocks was lower in E2 than E3 or E4 mice. In females, the response was also lower in the presence of hAPP (Figure 5D). When the freezing during the tones was analyzed, there was an effect of APP in males (Figure 5E) but not females (Figure 5F). In males, freezing levels were lower in NL-G-F/E3 and NL-G-F/E4 than E3 and E4 mice, respectively. This pattern not seen comparing NL-G/F/E2 and E2 mice.

Next we analyzed the freezing between the tone-shock intervals, a measure of fear learning. In males, there were effects of APP, APOE, and an APP × APOE interaction (Figure 5G). In females, there was only an APP × APOE interaction (Figure 5H). In males and females, NL-G-F/E2 mice showed more freezing between the tone-shock intervals than NL-G-F/E3 and NL-G-F/E4 mice. This pattern was not seen in the absence of hAPP. Actually, in males, E4 mice froze more than E2 or E3 mice between the tone-shock intervals.

The day after fear learning, we assessed hippocampus-dependent contextual fear memory. In males, there was an APP × APOE interaction (Figure 5I). NL-G-E2 mice showed more freezing during the context fear memory test than NL-G-F/E3 mice. This apoE genotype pattern was not seen in the absence of hAPP. In females, there was an effect of APOE with E2 mice freezing more during the context test than E3 mice.

Finally, we analyzed hippocampus-independent cued fear memory. When the freezing during the tone only was analyzed, there was an effect of APOE in males (Figure 5J) but not females (Figure 5K). Freezing during the tone in the cued fear memory test was lower in E2 than E3 or E4 mice. No significant effects on freezing during the tone were detected in females (Figure 5L). We also analyzed the freezing during the pre-tone and tone in the cued fear memory test as a repeated-measures ANOVA. In males, there was an effect of APOE with lower freezing levels in E2 than E4 mice (Figure 5K). This was sex-dependent and not seen in females (Figure 5L).

#### Cortical Aβ Levels

**FIGURE 6:**
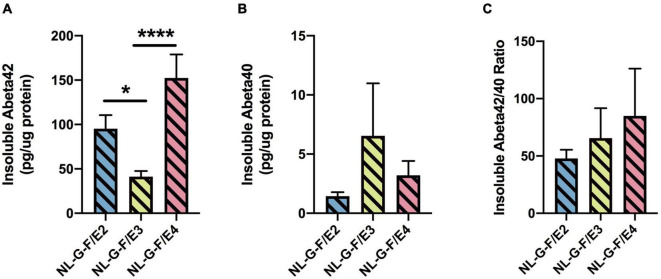
There was an effect of genotype on insoluble Aβ42 levels (*F* = 13. 83, *p* = 0.0002). Insoluble Aβ42 levels were lower in NL-G-F/E3 than NL-G-F/E2 (*p = 0.0170) and NL-G-F/E4 (^***^p < 0.0001) levels **(A)**. There were no significant genotype differences in insoluble Aβ40 levels **(B)** or the insoluble Aβ42/40 ratio **(C)**. Soluble Aβ42 levels were only detected in 2 NL-G-F/E2 mice (0.000698 and 0.000722 pg/μg protein), 3 NL-G-F/E3 mice (0.000148, 0.000593, and 0.00095 pg/μg protein), and 2 NL-G-F/E4 mice (0.001163 and 0.000637 pg/μg protein). Soluble Aβ40 levels were only detected in 2 NL-G-F/E2 mice (0.000183 and 9 × 10^– 5^ pg/μg protein) and 2 NL-G-F/E4 mice (0.000201 and 0.000387 pg/μg protein).

There was an effect of genotype on insoluble Aβ42 levels (*F* = 13. 83, *p* = 0.0002). Insoluble Aβ42 levels were lower in NL-G-F/E3 than NL-G-F/E2 (*p* = 0.0170) and NL-G-F/E4 (*p* < 0.0001) levels (Figure 6A). In addition, NL-G-F/E3 mice had lower cortical insoluble Aβ42 levels than NL-G-F/E2 mice. There were no significant genotype differences in insoluble Aβ40 levels (Figure 6B) or the insoluble Aβ42/40 ratio (Figure 6C). Soluble Aβ42 levels were only detected in 2 NL-G-F/E2 mice (0.000698 and 0.000722 pg/μg protein), 3 NL-G-F/E3 mice (0.000148, 0.000593, and 0.00095 pg/μg protein), and 2 NL-G-F/E4 mice (0.001163 and 0.000637 pg/μg protein). Soluble Aβ40 levels were only detected in 2 NL-G-F/E2 mice (0.000183 and 9 × 10^–5^ pg/μg protein) and 2 NL-G-F/E4 mice (0.000201 and 0.000387 pg/μg protein).

### Behavioral and Cognitive Performance of E2, E3, E4, NL-F/E2, NL-F/E3, and NL-F/E4 at the 18-Month Time Point

The results of the statistical analyses of the behavioral and cognitive performance at the 18-month time point are summarized in Supplementary Table 1B.

#### Body Weights

**FIGURE 7:**
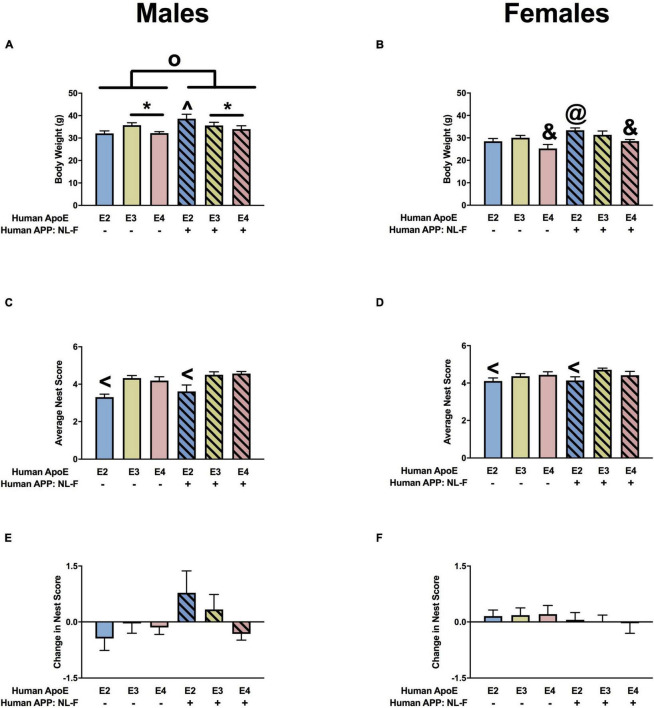
Body weights and nest score measures in males **(A,C,E)** and females **(B,D,F)** at the 18-month time point. **(A)** There was an effect of APOE and APP on body weights of males. E3 males were heavier than E4 males. *p < 0.05. In addition, NL-F males were heavier. *^o^p* < 0.05. NL-F/E2 mice were heavier than NL-F/E3 and E2 mice. *^o^p* < 0.05. **(B)** There was an effect of APOE and APP on body weights of females. E4 females were lighter than E2 and E3 females. ^&^*p* < 0.05. NL-F-E2 females were heavier than E2 females. ^@^*p* < 0.05. **(C)** There was an effect of APOE on nest scores of males. E2 males had lower nest scores than E3 and E4 males. ^<^*p* < 0.05. **(D)** There was a trend toward an effect of APOE on nest scores of females. E2 females had lower nest scores of E3 females. ^<^*p* < 0.05. **(E)** Change in nest score of males. **(F)** Change in nest scores of females.

For body weights, there were effects of APOE, APP, and an APP × APOE interaction in males (Figure 7A). Body weights were higher in NL-F/E2 than NL-F/E3 mice. This pattern was not seen in the absence of hAPP. Body weights were higher in E3 than E4 males. Body weights were also higher in the NL-F background. In females, there were also effects of APOE and APP on body weights (Figure 7B). Body weights were lower in E4 than E2 or E3 mice and were higher in the presence of NL-F.

#### Nest Building

There was an effect of APOE on nest building in males (Figure 7C). Nest building scores were lower in E2 than E3 or E4 males. There was a trend toward and effect of APOE on nest building in females (Figure 7D) but it did not reach significance. Nest building scores were lower in E2 than E3 females. There was a trend toward an effect of APP on the change in nest building scores in males (Figure 7E), but not females (Figure 7F).

#### Home Cage Activity

**FIGURE 8:**
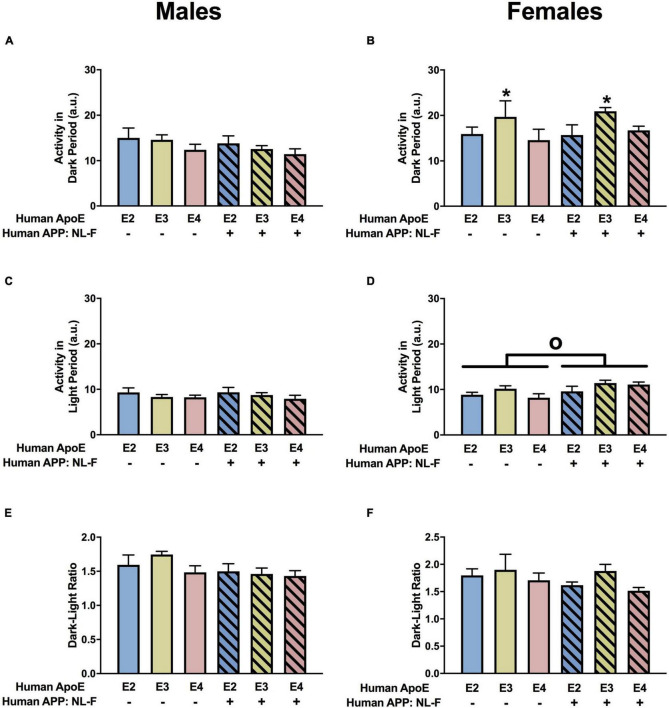
Home cage activity in males **(A,C,E)** and females **(B,D,F)** at the 18-month time point. **(A)** Activity of the males during the dark period. **(B)** There was an effect of APOE on activity levels of females during the dark period. E3 females were more active during the dark period than E2 and E4 females. *p < 0.05. **(C)** Activity of the males during the light period. **(D)** There was an effect of APP on activity of females during the light period. NL-F mice were more active during the light period. *^o^p* < 0.05. **(E)** There was a trend toward an effect of APP on the dark/light ratio of males, with a trend toward a lower dark/light ratio in NL/F males. **(F)** Dark/light ratio of females.

There was an effect of APOE on activity levels in the dark period in females (Figure 8B) but not males (Figure 8A. In females, E3 mice were more active during the dark period than E2 or E4 mice (Figure 8B). There was an effect of APP on activity levels during the light period in females (Figure 8D) but not males (Figure 8C). NL-F mice were more active during the light period. When the ratio of dark/light activity was analyzed, there were effects of APOE and APP and an APP × APOE interaction in males (Figure 8E). The dark/light ratio was lower in E4 than E2 or E3 mice and lower in NL-F mice than mice without hAPP. In females, there was an effect of APOE and an APP × APOE interaction for the dark/light ratio (Figure 8F). The dark/light ratio was also lower in E4 than E2 or E3 female mice and this effect was more pronounced in NL-G-F/E4 than E4 mice.

#### Elevated Zero Maze

**FIGURE 9:**
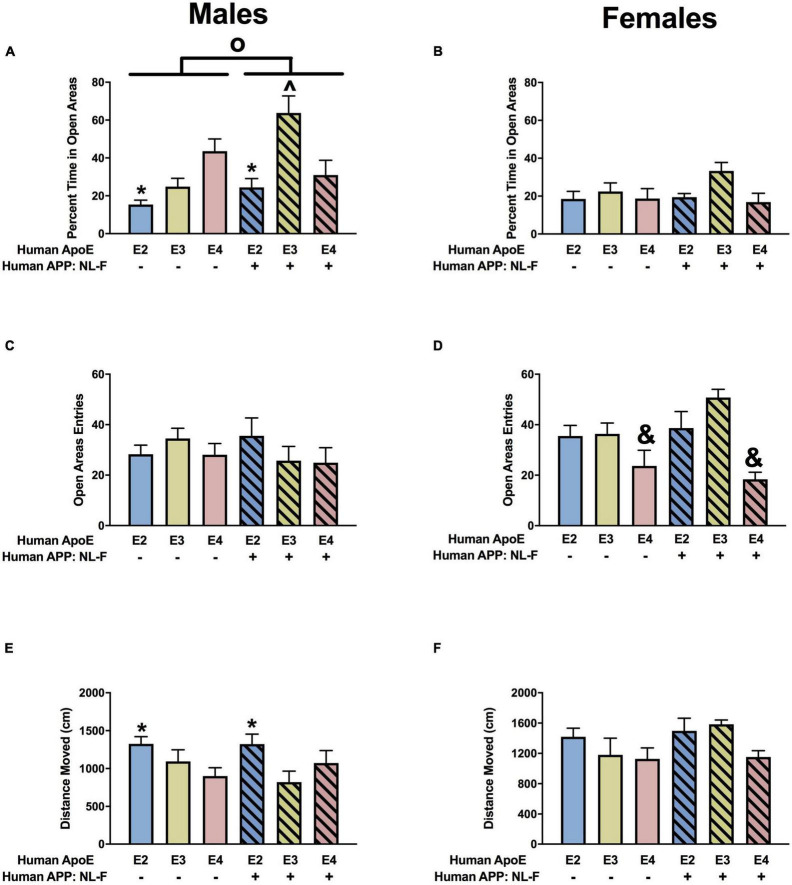
Performance of males **(A,C,E)** and females **(B,D,F)** in the elevated zero maze at the 18-month time point. **(A)** There was an effect of APOE, APP, and an APP × APOE interaction for percent time spent in the open areas in the elevated zero maze. E2 males showed higher measures of anxiety than E3 and E4 males. *p < 0.05. NL-F mice showed lower measures of anxiety. NL-F/E3 males showed lower measures of anxiety than NL-F/E4 and E3 males. ^*p* < 0.05. **(B)** There was a trend toward an effect of APOE on time spent in the open areas of the elevated zero maze in females, with a trend toward lower measures of anxiety in E3 than E4 females. **(C)** Entries of males into the open areas of the elevated zero maze. **(D)** There was an effect of APOE on entries into the open areas of the elevated zero maze. E4 females entered the open areas less than E2 and E3 mice. ^&^*p* < 0.05. **(E)** There was an effect of APOE on activity levels of males in the elevated zero maze. E2 males showed higher activity levels than E3 and E4 males. *p < 0.05. **(F)** Activity levels of females in the elevated zero maze.

When measures of anxiety were assessed in the elevated zero maze in males, there were effects of APOE, APP, and an APP × APOE interaction for the percent time spent in the open areas (Figure 9A). E2 males spent less time in the open areas than E3 and E4 males. In the presence of hAPP, the time spent in the open areas was less in E4 than E3 mice. However, in the presence of hAPP, the time spent in the open areas was less in NL-F/E3 than NL-F/E2 or NL-F/E4 mice. In addition, NL-F/E3 males spent more time in the open areas than E3 males. In females, there was a trend toward an effect of APOE on time spent in the open areas in the elevated zero maze (Figure 9B). We next analyzed entries into the more anxiety-provoking open areas of the elevated zero maze. There was an effect of APOE on entries into the open areas in females (Figure 9D) but not males (Figure 9C). E4 females entered the open areas less than E2 or E3 mice. There was an effect of APOE on activity levels in the elevated zero maze in males (Figure 9E) but not females (Figure 9F). In males, E2 mice were more active than E3 or E4 mice.

#### Wire Hang

**FIGURE 10:**
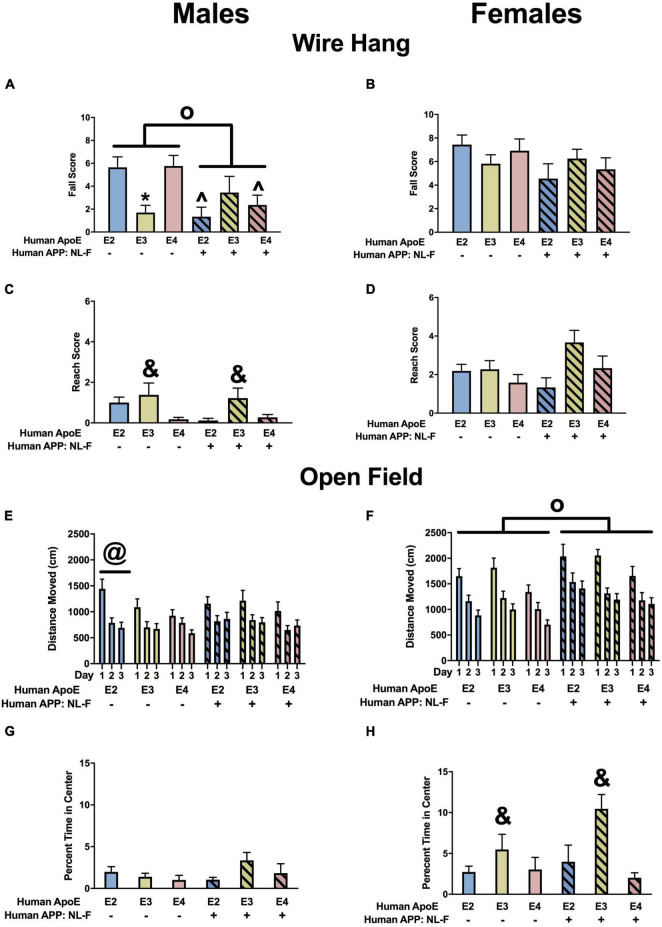
Performance of males **(A,C,E,G)** and females **(B,D,F,H)** in the wire hang and open field tests at the 18-month time point. **(A)** There was an effect of APP and an APP × APOE interaction for fall scores of males in the wire hang test. E3 males had lower fall scores than E2 and E4 males. *p < 0.05. NL-F/E2 and NL-F/E4 males had lower fall scores than E2 and E4 males, respectively. ^*p* < 0.05. N-L-F males had lower fall scores. *^o^p* < 0.05. **(B)** Fall scores of females in the wire hang test. **(C)** There was an effect of APOE on reach scores in the wire hang test. E3 males had higher reach scores than E4 males. ^&^*p* < 0.05. **(D)** There was a trend toward an effect of APOE on reach scores in the wire hang test. **(E)** There was an APP × APOE interaction for activity levels of males in the open field. E2 males were more active than E3 and E4 males on days 1 and 3 and E2 and E4 males were more active than E3 males on day 2. ^@^*p* < 0.05. **(F)** There was an effect of APP on activity of females in the open field. NL-F females were more active in the open field. *^o^p* < 0.05. **(G)** Percent time males spent in the center of the open field. **(H)** There was an effect of APOE on percent time females spent in the center of the open field. E3 females spent more time in the center of the open field than E2 and E4 females. ^&^*p* < 0.05.

There was an effect of APP and an APP × APOE interaction for fall scores in the males (Figure 10A). Fall scores were lower in NL-F/E2 and NL-F/E4 than E2 and E4 males mice, respectively. In addition, in the absence of NL-F, fall scores were lower in E3 than E2 or E4 males. In contrast to the males, there were no effects on fall scores in the females (Figure 10B). There was an effect of APOE on reach scores in males (Figure 10C). Reach scores were higher in E3 than E4 males. There was a trend toward an effect of APOE on reach scores in females but it did not reach significance (Figure 10D).

#### Open Field

When activity levels were assessed in the open field, there was an APP × apoE interaction in males (Figure 10E). In the absence of NL-F, E2 mice were more active than E3 mice. On days 1 and 3, E2 mice were also more active than E4 mice. On day 2, E4 mice were more active than E3 mice. In the E2 background, on day 1 E2 mice were more active than NL-F/E2 mice. However, on days 2 and 3 NL-F/E2 mice were more active than E2 mice. In females, there was an effect of APP and a trend toward an effect of apoE (Figure 10F). Activity levels were higher in the presence of NL-F.

Next measures of anxiety were assessed in the open field by analyzing the time spent in the more anxiety-provoking center of the open field. There was no effect of APP or apoE on percent time spent in the center of the open field in males (Figure 10G) but in females there was an effect of APOE and a trend toward an APP × apoE interaction (Figure 10H). E3 females spent more time in the open areas than E2 or E4 females. In the NL-F background, E3 female spent more time in the center of the open field than E4 females. Finally, NL-F/E3 females spent more time in the center of the open field than E3 females.

#### Y Maze

There was no effect of APP or apoE on spontaneous alternation in males (Supplementary Figure 2A). However, there was an APP × APOE interaction for spontaneous alternation in females (Supplementary Figure 2B). In the absence of NL-F, spontaneous alternation was lower in E3 than E4 females. In addition, spontaneous alternation was higher in NL-F/E3 than E3 females.

When activity levels were analyzed in the Y maze, there was an effect of APOE on the number of arm entries in males (Supplementary Figure 2C) and females (Supplementary Figure 2D). In males, activity levels were higher in E2 than E3 or E4 males (Supplementary Figure 2C). In females, activity levels were higher in E2 than E4 females (Supplementary Figure 2D).

#### Fear Conditioning

**FIGURE 11:**
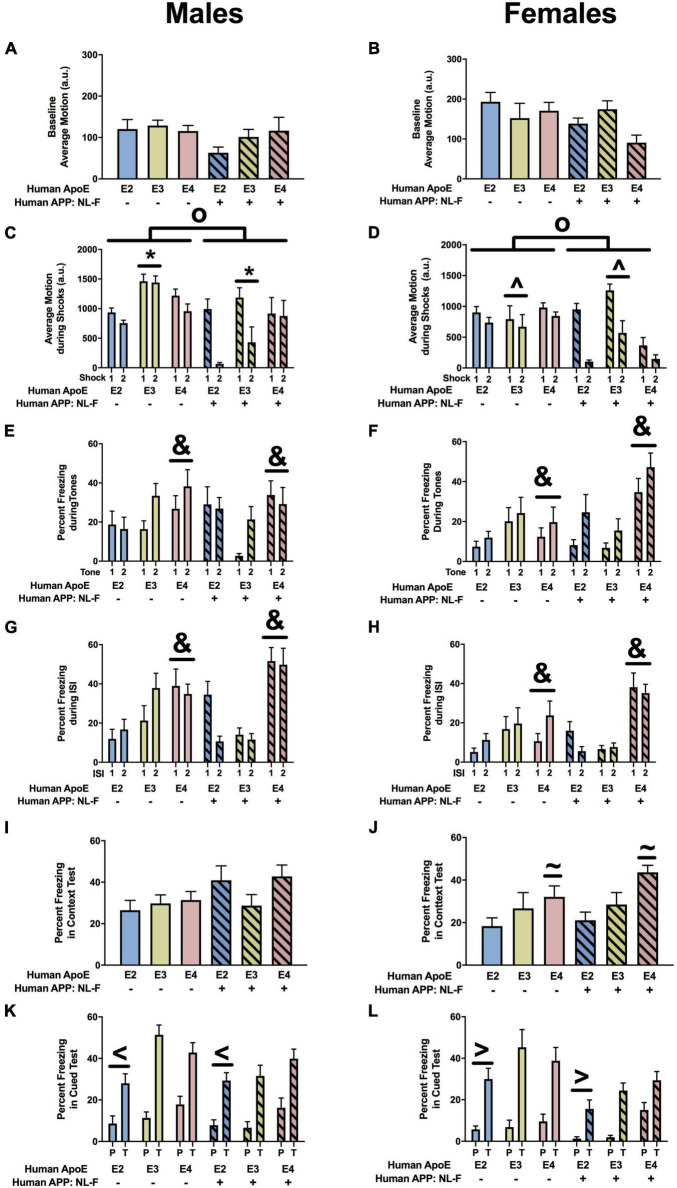
Performance of males **(A,C,E,G,I,K)** and females **(B,D,F,H,J,L)** in the fear conditioning test at the 18-month time point. **(A)** Baselines motion of males in the fear conditioning test. **(B)** There was a trend toward an effect of APP on baseline motion of females in the fear conditioning test. NL-F females showed lower activity levels during the baseline period. **(C)** There was an effect of APP, APOE, and an APP × APOE interaction for average motion of males during the shocks. E3 males moved more during the shocks than E2 males. *p < 0.05. In addition, NL-F males moved less during the shocks. *^o^p* < 0.05. **(D)** There was an effect of APP, APOE, and an APP × APOE interaction for average motion of females during the shocks. E3 females moved more during the shocks than E4 females. ^∧^*p* < 0.05. NL-F females also moved less during the shocks. *^o^p* < 0.05. **(E)** There was an effect of APOE on freezing of males during the tones. E4 males froze more during the tones than E2 and E3 males. ^&^*p* < 0.05. **(F)** There was an effect of APOE on freezing of females during the tones. E4 females froze more during the tones than E2 and E3 females. ^&^*p* < 0.05. **(G)** There was an effect of APP and trends toward effects of APOE and an APP × APOE interaction for freezing of males during the ISIs. E4 males froze more during the ISIs than E2 and E3 males. ^&^*p* < 0.05. **(H)** There was an effect of APP, APOE, and an APP × APOE interaction for freezing of females during the ISIs. E4 females froze more during the ISIs than E2 and E3 females. ^&^*p* < 0.05. **(I)** There was a trend toward an effect of APP on freezing of males during the contextual fear memory test. There was a trend toward higher freezing levels of NL-F males during the contextual fear memory test. **(J)** There was an effect of APOE on freezing levels of females during the contextual fear memory test. E4 females froze more than E2 females during the contextual fear memory test. ^&^*p* < 0.05. **(K)** There was an effect of APOE and a trend toward an effect of APP on freezing of males during the tone in the cued fear memory test. E2 males froze less than E3 and E4 males. ^<^*p* < 0.05. **(L)** There was an effect of APP and a trend toward an effect of APOE on freezing of females during the tone in the cued fear memory test. E4 females froze more during the tone than E2 females. ^>^*p* < 0.05.

There were no effects of APP or apoE on baseline motion in males (Figure 11A). There was a trend toward an effect of APP on baseline motion in females but it did not reach significance (Figure 11B). When response to the shocks was analyzed there was an effect of APOE, APP, and an APP × APOE interaction in males (Figure 11C) and females (Figure 11D). In males, E3 mice showed a stronger response to the shocks than E2 mice. In females, E3 mice showed a stronger response to the shocks than E4 mice. In addition, male and female mice showed a reduced response to the shocks in the NL-F background. In the NL-F background, E3 males showed a stronger response to the first shock than E2 and E4 males, while E4 males showed a stronger response to the second shock than E2 and E3 males. In the NLF background, E3 females showed a stronger response to both shocks compared to E2 and E4 females.

Next we analyzed freezing during the tones. There was an effect of APOE in males (Figure 11E) and females (Figure 11F). Freezing during the tones was higher in E4 than E3 or E2 mice. In females, there also was an effect of APP and an APP × APOE interaction (Figure 11F). In the absence of NL-F, freezing levels during the tones were higher in E3 than E2 than E4 females. Freezing levels during the tones were higher in the presence of NL-F. In contrast, freezing levels during the tones were higher in NL-F/E4 than NL-F/E2 or NL-F/E3 female mice.

When freezing between the tone-shock intervals was analyzed, there was an effect of APOE, APP, and an APP × APOE interaction in males (Figure 11G) and females (Figure 11H). E4 mice froze more during the tone-shock intervals than E2 and E3 mice. In males, in the mouse apoE background, E4 mice froze more during the tone-shock intervals than E2 mice. In females, NL-F/E4 mice from more during the tone-shock intervals than E4 mice.

In males, there was a trend toward an effect of APP on freezing in the contextual fear memory test but it did not reach significance (Figure 11I). In females, there was an effect of APOE on freezing during the contextual fear memory test (Figure 11J). Freezing levels in the contextual fear memory test were higher in E4 than E2 females.

In the cued fear memory test, when freezing during the tone only was analyzed, there was an effect of APOE in males (Figure 11K) and a trend toward an effect of APOE in females (Figure 11L). In males, E2 mice froze less during the tone than E3 and E4 mice. In addition, in there was an effect of APP in females (Figure 11L) and a trend toward an effect of APP in males (Figure 11K), with reduced cued fear memory in the NL-F background. When the pre-tone and tone were analyzed together, there was an effect of APOE in males and females, with lower freezing in E2 than E4 mice.

#### Cortical Aβ Levels

**FIGURE 12:**
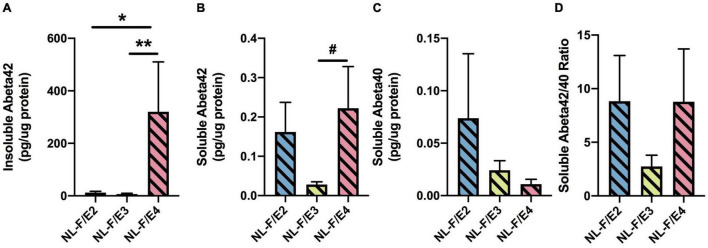
**(A)** Insoluble Aβ42 levels were higher in NL-F/E4 than NL-F/E2 and NL-F/E3 mice. *p = 0.0106, **** *p* = 0.0074. **(B)** There was a trend toward higher soluble Aβ42 levels in NL-F/E4 than NL-F/E3 mice. ^#^*p* = 0.0852. **(C)** There was no effect of genotype on soluble Aβ40 levels. **(D)** There was no effect of genotype on the soluble Aβ42/40 ratio.

There was an effect of genotype on insoluble Aβ42 levels (*F* = 5.873, *p* = 0.0090). Insoluble Aβ42 levels were higher in NL-F/E4 than NL-F/E2 (*p* = 0.0106) and NL-F/E3 (*p* = 0.0074) mice (Figure 12A). Insoluble Aβ40 levels could only be detected in 1 NL-F/E2 mouse (7.1 pg/μg protein), 2 NL-F/E3 mice (6.95 and 4.22 pg/μg protein) and 1 NL-F/E4 mouse (1.27 pg/μg protein). There was a trend toward an effect of genotype on soluble Aβ42 levels (*F* = 2.773, *p* = 0.0843), with a trend toward higher soluble Aβ42 levels in NL-F/E4 than NL-F/E3 mice (*p* = 0.0852, Figure 12B). There was no effect of genotype on soluble Aβ40 levels (Figure 12C) or the soluble Aβ42/40 ratio (Figure 12D).

#### Cortical Aβ Levels in NL-G-F, NL-F, and Wild-Type Mice at the 6-Month Time Point

**FIGURE 13:**
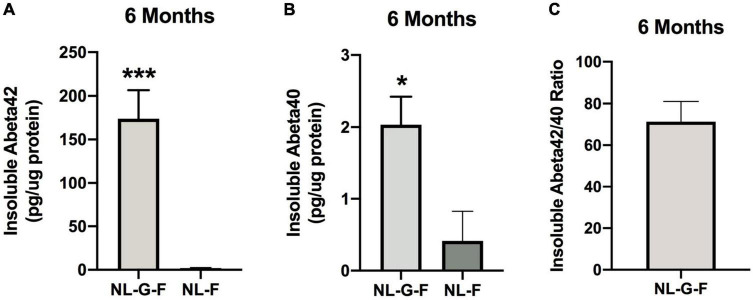
**(A)** Insoluble Aβ42 levels were higher in NL-G-F than NL-F mice. *** *p* < 0.0001. **(B)** Insoluble Aβ40 levels were higher in NL-G-F than NL-F mice. *p = 0.0279. **(C)** The insoluble Aβ42/40 ratio could only be calculated in NL-G-F mice.

The results of the statistical analyses of the behavioral and cognitive performance at the 6-month time point are summarized in Supplementary Table 1C. Insoluble Aβ42 levels were higher in NL-G-F than NL-F mice (*t* = 7.321, *p* < 0.0001, 2-sided) (Figure 13A). Insoluble Aβ40 levels were also higher in NL-G-F than NL-F mice (*t* = 2.571, *p* = 0.0279, 2-sided) (Figure 13B). The insoluble Aβ42/40 ratio could only be calculated in NL-G-F mice (Figure 13C). Soluble Aβ42 levels could not be detected in any NL-G-F or NL-F mice. Soluble Aβ40 levels could only be detected in one NL-G-F mouse (0000609 pg/μg protein).

### Behavioral and Cognitive Performance of NL-G-F, NL-F, and Wild-Type Mice at the 18-Month Time Point

#### Body Weights

There was an effect of APP on body weights in males, with lower body weights in NL-F than NL-G-F males (Supplementary Figure 3A). This APP effect was sex dependent and not seen in females (Supplementary Figure 3B).

#### Nest Building

There was no effect of APP on nest scores or in the change in nest building scores in females or males.

#### Home Cage Activity

There was a trend toward an effect of APP on activity during the dark period in males, with NL-G-F males being more active than WT males but it did not reach significance (Supplementary Figure 3C). This trend was not seen in females (Supplementary Figure 3D). There was also an effect of APP on activity during the light period in females (Supplementary Figure 3F), but not in males (Supplementary Figure 3E). There was a trend toward less activity of WT than NL-F and NL-G-F females during the light period.

#### Elevated Zero Maze

There was no effect of APP on measures of anxiety in the elevated zero maze in males or females. There was an effect of APP on entries into the open areas in the elevated zero maze in males and females. NL-F males entered the open areas more than WT males. There was also a trend toward NL-G-F males entering the open areas more than WT males but it did not reach significance. In females, NL-G-F mice entered the open areas more than NL-F and WT mice.

#### Wire Hang

There was an effect of APP on fall scores in males (Supplementary Figure 3G) and females (Supplementary Figure 3H). In males and females, NL-G-F mice had lower fall scores than NL-F and WT mice.

There was an effect of APP on reach scores in males (Supplementary Figure 3I) but not females (Supplementary Figure 3J). NL-G-F males had lower reach scores than NL-F males. In addition, there was a trend toward lower reach scores in WT than NL-F males but that did not reach significance.

#### Open Field

There was no effect of APP on activity levels during the 3 days of open field testing in males (Supplementary Figure 3K) and females (Supplementary Figure 3L).

There was an effect of APP on time spent in the center of the open field in males but not females. In males, the WT mice spent less time in the center of the open field than NL-G-F and NL-F mice.

#### Fear Conditioning

There was an effect of APP on baseline activity (prior to the first tone-shock pairing) in males (Supplementary Figure 4A), with higher baseline activity in NL-F than WT and NL-G-F males. This was sex-dependent and not seen in females (Supplementary Figure 4B).

There were no effects of APP on percent freezing during the tones, response to the shock, or freezing between the tone-shock periods in males or females.

There was an effect of APP on percent freezing of males (Supplementary Figure 4C) and females (Supplementary Figure 4D) in the contextual fear memory test. In males and females, NL-G-F mice froze less in the contextual fear memory test than WT and NL-F mice.

There was also an effect of APP on percent freezing during the cued fear memory test in males (Supplementary Figure 4E) and females (Supplementary Figure 4F), when freezing during the tone alone was analyzed. In males, NL-G-F mice froze less than WT or NL-F mice. In females, NL-G-F mice froze less than NL-F mice.

#### Cortical Aβ Levels in NL-G-F, NL-F, and Wild-Type Mice at the 18-Month Time Point

**FIGURE 14:**
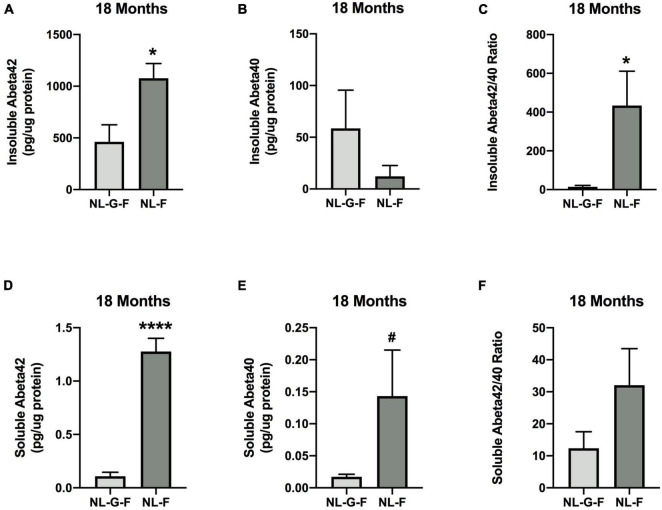
**(A)** Insoluble Aβ42 levels were higher in NL-F than NL-G-F mice. *p = 0.0116. **(B)** Insoluble Aβ40 levels were not different between the two lines. **(C)** The insoluble Aβ42/40 ratio was higher in NL-F than NL-G-F mice. *p = 0.0171. **(D)** Soluble Aβ42 levels were higher in NL-F than NL-G-F mice. ****p < 0.0001. **(E)** There was a trend toward higher soluble Aβ40 levels in NL-F than NL-G-F mice. ^#^*p* = 0.0822. **(F)** There was no line difference in the soluble Aβ42/40 ratio.

Insoluble Aβ42 levels were higher in NL-F than NL-G-F mice (*t* = 2.828, *p* = 0.0116, 2-sided) Figure 14A). Insoluble Aβ40 levels were not different between these two lines (Figure 14B). The insoluble Aβ42/40 ratio was also higher in NL-F than NL-G-F mice (*t* = 2.767, *p* = 0.0171, 2-sided) (Figure 14C).

Soluble Aβ42 levels were higher in NL-F than NL-G-F mice (*t* = 8.649, *p* < 0.0001, 2-sided) (Figure 14D). There was a trend toward higher soluble Aβ40 levels in NL-F than NL-G-F mice (*t* = 1.883, *p* = 0.0822, 2-sided) (Figure 14E). There was no difference in the soluble Aβ42/40 ratio (Figure 14F).

#### Relationships Between Insoluble Cortical Aβ Levels and Behavioral Measures in 6-Month-Old NL-G-F and 18-Month-Old NL-F Mice

**FIGURE 15:**
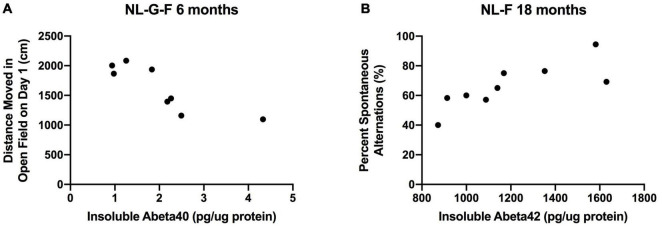
**(A)** At 6 months of age, insoluble cortical Aβ levels were negatively correlated with activity levels on the first day of open field testing (*r* = –0.8494, *p* = 0.0076, *n* = 8 XY pairs). **(B)** At 18 months of age, insoluble cortical Aβ42 levels were positively correlated with percent spontaneous alternation in the Y maze (*r* = 0.7943, *p* = 0.0106, *n* = 9 XY pairs).

Finally, we assessed whether insoluble cortical Aβ levels correlated with behavioral measures in 6-month-old NL-G-F mice and 18-month-old NL-F mice. At 6 months of age, insoluble cortical Aβ40 levels were negatively correlated with activity levels on the first day of open field testing (*r* = −0.8494, *p* = 0.0076, *n* = 8 XY pairs, Figure 15A). A similar but less robust relationship was seen on the second day of open field testing (*r* = −0.7394, *p* = 0.036, *n* = 8 XY pairs, Figure 15A).

At 18 months of age, insoluble cortical Aβ42 levels were positively correlated with percent spontaneous alternation in the Y maze (*r* = 0.7943, *p* = 0.0106, *n* = 9 XY pairs, Figure 15B).

## Discussion

The results of this study show that apoE has isoform-dependent effects on hAPP/Aβ-induced behavioral alterations and cognitive impairments and insoluble cortical Aβ42 levels in adult female and male mice at 6 and 18 months of age. Supplementary Table 1D illustrates all behavioral measures that showed APP × APOE interactions, supporting that apoE has isoform-dependent effects on hAPP/Aβ-induced behavioral and cognitive performance. While NL-G-F/E3, but not NL-G-F/E2, mice had lower cortical insoluble Aβ42 levels than NL-G-F/E4 mice. NL-F/E3 and NL-F/E2 mice had lower cortical insoluble Aβ42 levels than NL-F/E4 mice. Insoluble cortical Aβ42 and Aβ40 levels were higher in 6-month-old NL-G-F than NL-F mice. At 18 months of age, insoluble and soluble cortical Aβ42 and the insoluble cortical Aβ42/40 ratio were higher in NL-F than NL-G-F mice.

At 6 months of age, average nest score, and fear learning (percent freezing during the ISIs) showed APP × APOE interactions in males and females. Other measures showed sex-dependent APP × APOE interactions at 6 months of age. Change in nest score, entries in the open areas of the elevated zero maze, and contextual fear memory showed APP × APOE interactions in males. In contrast, average body weight, average nest score, activity during the light period, percent time in the open areas of the elevated zero maze, activity levels in the elevated zero maze, reach scores in the wire hang test, and baseline motion in the fear conditioning test showed APP × APOE interactions in females. At 18 months of age, average motion during the shocks during fear learning, and percent freezing during the ISIs showed APP × APOE interactions in males and females. Thus, at both 6 and 18 months an APP × APOE interaction is seen for fear learning in both sexes. Other measures showed sex-dependent APP × APOE interactions at 18 months of age. Average body weight, percent time in the open areas of the elevated zero maze, wire hang fall score, activity levels in the open field, showed APP × APOE interactions in males. In contrast, percent time in the center of the open field, percent spontaneous alternation in the Y maze, and percent freezing during the tones in the fear conditioning test showed APP × APOE interactions in females. While in males, measures of anxiety in the open field revealed an APP × APOE interaction at the 6-month time point, measures of anxiety in the elevated zero maze revealed an APP × APOE interaction at the 18-month time point. The opposite pattern was seen in females. In males, measures of anxiety in the elevated zero maze revealed an APP × APOE interaction at the 6-month time point, measures of anxiety in the open field revealed an APP × APOE interaction at the 18-month time point. Thus, although the measures of anxiety in the elevated zero maze and open field are distinct, they revealed an APP × APOE interaction at 6 and 18 months in males and females and highlight the importance of including more than one test to assess measures of anxiety. We recognize that age-related changes in performance in the behavioral tests used in this study, and the differential effects of the NL-G-F and NL-F mutation might have contributed to the distinct behavioral measures revealing an APP × APOE interaction at these two time points. The fact that at both 6 and 18 months an APP × APOE interaction is seen for fear learning in both sexes is remarkable considering age-related changes in hearing and responsiveness to aversive challenges and age-related changes in fear learning in fear conditioning paradigms in humans (Labar et al., 2004) and rodents. However, this result is consistent with the small or no age-related difference in fear learning reported in wild-type mice (for a review, see Foster et al., 2012).

For body weights of females at 6 months age, NL-G-F/E4 mice were heavier than E4 mice. In contrast, NL-G-F/E2 and NL-G-F/E3 mice were lighter than E2 and E3 mice, respectively. For body weights of males at 18 months of age, NL-F/E2 mice were heavier than E2 mice and no differences in body weight were seen in the E3 and E4 background. The observation that these changes occurred in females at 6 months of age but in males at 18 months of age is consistent with the human data showing that while changes in body mass index within 2 years increased AD risk in women, changes in body mass over 4 years were required to increase AD risk in men (Kang et al., 2021). With regard to the direction of the body weight changes seen, it is hard to conclude whether a gain or loss in body weight would be more detrimental as both increases and decreases in body mass are associated with increased AD risk (Park et al., 2019).

At 6 months age, for average nest scores in males and females and for change in nest scores in males, NL-G-F/E2 mice had higher scores than E2 mice. This was genotype-dependent and not seen in the E3 or E4 background. As nest scores might reflect activities of daily living which are negatively impacted in AD (Prizer and Zimmerman, 2018) and increased measures would be expected to be beneficial, these data suggest a beneficial effect of NL-G-F in the E2 background.

When percent time spent in the anxiety-provoking areas of the elevated zero maze were analyzed in females at 6 months of age, measures of anxiety were lower in NL-G-F/E2 than E2 mice. This effect was genotype-dependent and not seen in the E3 and E4 background. At the 18-month time point, NL-F/E3 male mice were less anxious than E3 male mice and there was a trend toward lower measures of anxiety in NL-F/E2 than E2 male mice. These data suggest a beneficial effects of NL-F in the E2 and E3 backgrounds. In addition, NL-F/E3 males showed lower measures of anxiety than NL-F/E4 males in the elevated zero maze and NL-F/E3 females showed lower measures of anxiety than NL-F/E4 females in the open field. These data suggest a beneficial effect of E3 in the NL-F background. These effects are translationally relevant, as increased anxiety levels are observed in patients with mild cognitive impairment, mild dementia, or early-onset AD, and can promote progression or conversion to AD (Mendez, 2021).

At the 6-month time point, there was less fear learning in NL-G-F/E4 than E4 males. There was a trend toward such effect in the E3 background. However, this was not seen in the E2 background. In addition, E2 males showed better fear learning than E3 or E4 males. Contextual fear memory was also stronger in NL-G-F/E2 than NL-G-F/E3 mice. Finally, NL-G-F/E3 males showed less contextual fear memory than E3 males. These effects are consistent with a protective effect of E2 against a NL-G-F-induced learning deficit in males. In females, this effect was less clear; only during the second ISI was there less fear learning in NL-G-F/E4 than E4 females. In contrast to what was seen at the 6-month time point in E4, at the 18-month time point NL-F/E4 female mice showed better fear learning than E4 female mice and NL-F/E4 male and female mice showed more fear learning than sex-matched NL-F/E3 or NL-F/E2 mice. In addition, NL-F/E4 female mice froze more during the tones during the training day than NL-F/E3 and NL-F/E2 female mice. These data indicate that NL-F enhances fear learning in a sex-dependent fashion.

For spontaneous alternation in Y maze at the 18-month time point, we did not see a detrimental effect of NL-F or E4. In females, E4 mice showed more spontaneous alternation than E3 mice. In addition, NL-F/E3 females showed more spontaneous alternation than E3 females and no effects of NL-F on spontaneous alternation of E2 or E4 females. We did not see an effect of APP on spontaneous alternation in NL-G-F, NL-F, and WT male or female mice at the 18-month time point either. At the 6-month time point, we did report a genotype effect of APP on spontaneous alternation in females with lower spontaneous alternation in NL-G-F than WT females (Kundu et al., 2021). In contrast, we did not see an effect of APP on spontaneous alternation in males (Kundu et al., 2021). We recognize that reduced spontaneous alternation was reported in 18-month-old NL-F males and 6-month-old NL-G-F males (Saito et al., 2014). While we used the same model Y maze as used in those studies, different environmental conditions might have contributed to those divergent findings.

For activity levels on second and third day in the open field and the 18-month time point, NL-F/E2 males moved more than E2 males. These data suggest reduced hippocampus-dependent spatial habituation learning in the NL-F background. E2 males moved more than E3 males on all days, and E3 males moved than E4 males on days 1 and 3. Based on the association of low levels of physical activity with AD risk and the beneficial effects of physical exercise on the prevention and treatment of AD (De la Rossa et al., 2020), these results suggest that some of the beneficial effects of E2 might be related to enhanced activity levels during aging. However, when wire hang fall scores were analyzed at the 18-month time point, NL-F/E2 and NL-F/E4 males showed lower scores than E2 and E4 males, while there was no effect of NL-F on wire hang fall scores of E3 males. These data indicate that the differential apoE isoform-dependent effects of NL-F on behavioral measures might not relate to differences in muscle strength or coordination.

Finally, we compared behavioral and cognitive performance of 18-month-old *App^NL–G–F^* and *App^NL–F^* female and male mice on a murine apoE background along with that of age—and sex-matched C57BL/6J wild-type mice. Consistent with a worse cognitive phenotype of NL-G-F than NL-F mice, NL-G-F females showed less contextual fear memory than WT or NL-F females. This cognitive impairment was not restricted to females or contextual fear memory. NL-G-F males showed less cued fear memory than NL-F or WT males and NL-G-F females showed less cued fear memory than NL-F females. These effects did not seem related to possible differences in baseline activity levels. NL-F males showed higher baseline activity levels (prior to the first tone-shock pairing) than NL-G-F and WT males. In the wire hang test, NL-G-F males had lower fall scores than NL-F and WT males. NL-G-F males also had lower reach scores than NL-F males. NL-G-F females had lowed fall scores than NL-F and WT females. These effects did not seem related to possible differences in anxiety levels either. In the elevated zero maze, NL-F males entered the open areas more often than WT males and there was a trend toward NL-G-F males entering the open areas more often than WT males. In addition, NL-G-F and NL-F males spent more time in the center of the open field than WT males. Similarly, NL-G-F females entered the open areas of the elevated zero maze more often the NL-F or WT females.

In females, there was an effect of APP on activity during the light period, with a trend toward higher activity levels in NL-G-F and NL-F than WT females. This might be related to sleep disturbances in AD (Brzecka et al., 2018) and the interaction between sleep disturbances and AD and brain function (Li et al., 2019).

At 6 months of age, prior to the onset of plaque pathology, E3, but not E4, was able to protect against hAPP/Aβ-induced impairments in spatial memory retention in the water maze in a mouse model in which hapoE was not expressed outside the brain and E3 or E4 was not expressed under control of an apoE promotor in the brain and expressed at similar levels (Raber et al., 2000). Similarly, E3 protected against hAPP/Aβ-induced neuropathology in these mouse models (Holtzman et al., 1999; Raber et al., 2000; Hartman et al., 2001; Buttini et al., 2002; Fagan et al., 2002). These data suggest that compared to E3, E4 is detrimental whether expressed in brain at similar levels as E3 or not and whether the apoE and APP genes are knockins or transgenically expressed.

NL-G-F/E3, but not NL-G-F/E2, mice had lower cortical insoluble Aβ42 levels than NL-G-F/E4 mice. In addition, NL-G-F/E3 mice had lower cortical insoluble Aβ42 levels than NL-G-F/E2 mice. In contrast, both NL-F/E3 and NL-F/E2 mice had lower cortical insoluble Aβ42 levels than NL-F/E4 mice. These data suggest that the protective effect of E2 on Aβ42 pathology in aged mice in the APP KI model is age-dependent and not seen earlier in life. In contrast to these data and this model, in hapoE TR mice crossed with hAPP and presinilin 1 (PS1) with exon 9 deletion overexpressing mice (APP_SW_/PS1_dE9_), containing both human and murine APP, there was a greater load of Aβ pathology in hAPP/E4 mice than APP/E2 mice at 6–10 months of age (Pankiewicz et al., 2014) or 11 months of age (Kuszczyk et al., 2013). These data suggest that the dramatic beneficial effects of E2 as compared to E4 on Aβ42 pathology in adult to middle-aged mice might more pronounced in mice expressing both human APP and presinilin and murine APP. These data are intriguing considering the strong protective effect of E2 with a later age at AD onset and lower levels of Aβ plaques and tau tangles in those carrying two copies of *APOE* ε2 or one copy of *APOE* ε2 and one copy of *APOE* ε3 (Reiman et al., 2020) and the beneficial effects of the *APOE3* Christchurch (R136S) mutation reported in an individual who carried a PS1 mutation (Arboleda-Velasquez et al., 2019). Considering the increased risk of those with E2 to develop more severe symptoms of post-dramatic stress disorder and/or an increased risk to develop (PTSD) (Olsen et al., 2012; Johnson et al., 2015), increased efforts are warranted to study the age-dependency of E2 effects on brain function.

While at 6 months of age, insoluble cortical Aβ42 and Aβ40 levels were higher NL-G-F than NL-F mice, at 18 months of age insoluble and soluble cortical Aβ42 and the insoluble cortical Aβ42/40 ratio were higher in NL-F than NL-G-F mice. Remarkably, in 6-month-old NL-G-F mice insoluble cortical Aβ40 levels were negatively related to activity in the open field in the absence and presence of objects. In contrast, at 18 months of age, insoluble cortical Aβ42 levels in NL-F mice were positively correlated with cognitive performance in the Y maze. These data suggest that the Aβ species and age might modulate the relationship between Aβ pathology and behavioral measures, consistent with the independent association of Aβ pathology and hippocampal atrophy with memory seen in cognitively healthy elderly (Svenningsson et al., 2019) and the baseline cognitive performance predicting progression to Aβ positivity in nondemented elderly (Elman et al., 2020).

In summary, these data support that there are apoE isoform-dependent effects on hAPP/Aβ-induced behavioral alterations and cognitive impairments and Aβ pathology in mouse models containing only human APP and apoE and not the murine counterparts. Future efforts are warranted to identify and compare the mechanisms underlying these apoE isoform-dependent effects in adulthood and aging.

## Data Availability Statement

The raw data supporting the conclusions of this article will be made available by the authors, without undue reservation.

## Ethics Statement

The animal study was reviewed and approved by OHSU IACUC.

## Author Contributions

JR designed the experiments. ET and SH supervised the breeding and the related genotyping. DG developed the genotyping protocols. DG, DK, and SH genotyped the samples. PK and SH performed the behavioral testing and related analyses. SH performed the statistical analyses for the behavioral data and helped with the related tables. RS helped with organizing and graphing of all the behavioral data. JR and SH processed the cortical samples for the Aβ ELISAs and analyzed the related data. All authors contributed to the writing of this manuscript.

## Conflict of Interest

The authors declare that the research was conducted in the absence of any commercial or financial relationships that could be construed as a potential conflict of interest.

## Publisher’s Note

All claims expressed in this article are solely those of the authors and do not necessarily represent those of their affiliated organizations, or those of the publisher, the editors and the reviewers. Any product that may be evaluated in this article, or claim that may be made by its manufacturer, is not guaranteed or endorsed by the publisher.

## Supplementary Material

Supplementary Figure 1 Performance of males **(A,C)** and females **(B,D)** in the Y maze at the 6-month time point. **(A)** Spontaneous alternation of males at the 6-month time point. **(B)** Spontaneous alternation of females at the 6-month time point. **(C)** There was an effect of APOE and APP on activity levels of males in the elevated zero maze. E2 males were more active in the elevated zero maze than E3 and E4 males. *^o^p* < 0.05. In addition, NL-G-F mice were less active. ^*p* < 0.05. **(D)** There also was an effect of APOE and APP on activity levels of females in the elevated zero maze. E2 females were more active in the elevated zero maze than E4 females. ^&^*p* < 0.05. In addition, NL-G-F mice were less active. ^*p* < 0.05.

Supplementary Figure 2 Performance of males **(A,C)** and females **(B,D)** in the Y maze at the 18-month time point. **(A)** Spontaneous alternation of males at the 18-month time point. **(B)** There was an APP × APOE interaction for spontaneous alternation of females at the 18-month time point. E4 females showed more spontaneous alternation than E3 females. **p* < 0.05. In addition, NL-F/E3 females showed more spontaneous alternation than E3 females. ^*p* < 0.05. **(C)** There was an effect of APOE on activity levels of males in the Y maze. Activity levels were higher in E2 than E3 and E4 males. ^&^*p* < 0.05. **(D)** There was an effect of APOE on activity levels of females in the Y maze. Activity levels were higher in E2 than E4 females. ^@^*p* < 0.05.

Supplementary Figure 3 Body weights, home cage activity, performance in the wire hang, and open field of male **(A,C,E,G,I,K)** and female **(B,D,F,H,J,L)** NL-G-F, NL-F, and WT mice at the 18-month time point. **(A)** There was an effect of APP on body weights of males. NL-G-F males were heavier than NL-F males. **p* < 0.05. **(B)** Body weights of NL-G-F, NL-F, and WT female mice. **(C)** There was a trend toward an effect of APP on activity of males during the dark period. There was a trend toward higher activity levels in NL-G-F than WT males. **(D)** Activity levels of NL-G-F, NL-F, and WT females during the dark period. **(E)** Activity levels of NL-G-F, NL-F, and WT males during the light period. **(F)** There was an effect of APP on activity levels of females during the light period. There was a trend of NL-F and NL-G-F females being more active during the light period than WT females. **(G)** There was an effect of APP on fall scores of males in the wire hang test. NL-G-F males had lower fall scores than NL-F and WT males. **p* < 0.05. **(H)** There was an effect of APP on fall scores of females in the wire hang test. NL-G-F females had lower fall scores than NL-F and WT females. **(I)** There was an effect of APP on reach scores of males in the wire hang test. NL-G-F males had lower reach scores than NL-F males. ^*p* < 0.05. **(J)** Reach scores of NL-G-F, NL-F, and WT females. **(K)** Activity levels of NL-G-F, NL-F, and WT males in the open field. **(L)** Activity levels of NL-G-F, NL-F, and WT females in the open field.

Supplementary Figure 4 Performance of NL-G-F, NL-F, and WT males **(A,C,E)** and females **(B,D,F)** at the 18-month time point. **(A)** There was an effect of APP on baseline motion of the males. NL-F males moved more during the baseline period than NL-G-F and WT males. **p* < 0.05. **(B)** Activity levels of NL-G-F, NL-F, and WT females during the baseline period. **(C)** There was an effect of APP on contextual fear memory of males. NL-G-F males froze less than NL-F and WT males during the contextual fear memory test. *^o^p* < 0.05. **(D)** There was an effect of APP on contextual fear memory of females. NL-G-F females also froze less than NL-F and WT females during the contextual fear memory test. *^o^p* < 0.05. **(E)** There was an effect of APP on cued fear memory of males. NL-G-F males froze less during the tone than NL-F and WT males. *^o^p* < 0.05. **(F)** There was an effect of APP on cued fear memory of females. NL-G-F females froze less during the tone than NL-F females. ^*p* < 0.05.
