# Supplementary material for: Apolipoprotein E Isoform-Dependent Effects on Human Amyloid Precursor Protein/Aβ-Induced Behavioral Alterations and Cognitive Impairments and Insoluble Cortical Aβ42 Levels
Source: Front Aging Neurosci. 2022 Mar 1;14:767558. doi: 10.3389/fnagi.2022.767558 (PMC8922030; doi:10.3389/fnagi.2022.767558)
Supplement: Supplementary file 1 [file Data_Sheet_1.docx]

**Suppl. Table 1A.** Statistical analyses of behavioral performance of mice tested at the 6-month time point ^1,2,3^.

| *Behavioral Measure* | *Sex* | *Effect of APOE* | *Direction of APOE effect ^5^* | *Effect of APP (NL-G-F* | *Direction of APP effect* | *APP x APOE interaction* | *Direction of effect in mAPP* | *Direction of effect in APP NL-G-F* | *Direction of effect in each APOE genotype* |
| --- | --- | --- | --- | --- | --- | --- | --- | --- | --- |
| Average Body Weight | M | *F* (2, 77) = 16.174, *p* < 0.001 | E2 > E3 & E4 | *F* (1, 77) = 5.516, *p* = 0.021 | mAPP > NL-G-F |  |  |  |  |
| Average Body Weight | F | *F* (2, 66) = 30.486, *p* < 0.001 | E2 > E3 & E4 | *F* (1, 66) = 6.234, *p* = 0.015 | mAPP > NL-G-F | *F* (2, 66) = 9.518,  *p* < 0.001 | E2 > E3 & E4 | E2 > E3 & E4 | E2: mAPP > NL-G-F  E3: mAPP > NL-G-F  E4: NL-G-F > mAPP |
| Average Nest Score | M | *F* (2, 87) = 18.225, *p* < 0.001 | E2 < E3 & E4 | *F* (1, 87) = 21.110, *p* < 0.001 | mAPP < NL-G-F | *F* (2, 87) = 16.053, *p* < 0.001 | E2 < E3 & E4 | ns | E2: NL-G-F > mAPP  E3: ns  E4: ns |
| Average Nest Score | F | *F* (2, 80) = 4.604, *p* = 0.013 | E2 < E3 & E4 | *F* (1, 80) = 6.403, *p* = 0.013 | mAPP < NL-G-F | *F* (2, 80) = 15.618, *p* < 0.001 | E2 < E3 & E4 | ns | E2: NL-G-F > mAPP  E3: ns  E4: ns |
| Change in Nest Score | M | *F* (2, 87) = 2.937, *p* = 0.058 | E4 > E3 |  |  | *F* (2, 87) = 5.735,  *p* = 0.005 | E4 > E2 | E3 < E2 & E4 | E2: NL-G-F > mAPP  E3: ns  E4: ns |
| Change in Nest Score | F |  |  |  |  |  |  |  |  |
| Activity in the Dark Cycle | M |  |  |  |  | *F* (2, 59) = 3.105,  *p* = 0.052 | E3 > E4 | ns | E2: mAPP < NL-G-F  E3: ns  E4: ns |
| Activity in the Dark Cycle | F |  |  |  |  | *F* (2, 64) = 2.642,  *p* = 0.079 | E2 < E3 & E4 | ns | E2: ns  E3: ns  E4: ns |
| Activity in the Light Cycle | M |  |  | *F* (1, 59) = 14.514, *p* < 0.001 | NL-G-F > mAPP |  |  |  |  |
| Activity in the Light Cycle | F |  |  | *F* (1, 64) = 4.268, *p* = 0.043 | NL-G-F > mAPP | *F* (2, 64) = 4.633,  *p* = 0.013 | E2 < E3 & E4 | ns | E2: mAPP < NL-G-F  E3: ns  E4: ns |
| Activity Ratio Dark/Light | M | *F* (2, 59) = 6.729, *p* = 0.002 | E3 > E2 | *F* (1, 59) = 17.363, *p* < 0.001 | mAPP > NL-G-F |  |  |  |  |
| Activity Ratio Dark/Light | F |  |  |  |  |  |  |  |  |
| Elevated Zero Maze: % Time in the Open Areas | M |  |  | *F* (1, 87) = 4.501, *p* = 0.037 | NL-G-F > mAPP |  |  |  |  |
| Elevated Zero Maze: % Time in the Open Areas | F |  |  | *F* (1, 80) = 5.402, *p* = 0.023 | NL-G-F > mAPP | *F* (2, 80) = 3.919,  *p* = 0.024 | ns | ns | E2: NL-G-F > mAPP  E3: ns  E4: ns |
| Elevated Zero Maze: Entries in Open Areas | M |  |  |  |  | *F* (2, 87) = 3.752,  *p* = 0.027 | ns | E3 > E2 & E4 | E2: ns  E3: NL-G-F > mAPP  E4: ns |
| Elevated Zero Maze: Entries in Open Areas | F | *F* (2, 80) = 3.931, *p* = 0.024 | E3 > E2 & E4 | *F* (1, 80) = 4.383, *p* = 0.039 | NL-G-F > mAPP |  |  |  |  |
| Elevated Zero Maze: Total Distance Moved (cm) | M | *F* (2, 87) = 2.770, *p* = 0.068 | E3 > E4 |  |  |  |  |  |  |
| Elevated Zero Maze: Total Distance Moved (cm) | F |  |  |  |  | *F* (2, 86) = 3.342,  *p* = 0.040 | ns | E3 < E2 | E2: ns  E3: NL-G-F > mAPP  E4: ns |
| Wire Hang: Fall Score | M | *F* (2, 87) = 3.532, *p* = 0.034 | E3 < E2 & E4 |  |  |  |  |  |  |
| Wire Hang: Fall Score | F | *F* (2, 80) = 3.207, *p* = 0.046 | E2 > E3 |  |  |  |  |  |  |
| Wire Hang: Reach Score | M | *F* (2, 87) = 6.699, *p* = 0.002 | E3 > E2 & E4 | *F* (1, 87) = 5.760, *p* = 0.019 | NL-G-F > mAPP |  |  |  |  |
| Wire Hang: Reach Score | F |  |  | *F* (1, 80) = 5.962, *p* = 0.017 | NL-G-F > mAPP | *F* (2, 80) = 5.374, *p* = 0.006 | E3 > E4 | ns | E2: NL-G-F > mAPP  E3: ns  E4: NL-G-F > mAPP |
| Open Field: Total Distance Moved (cm) ^4^ | M | *F* (2, 87) = 5.167, *p =* 0.008 ^2^ | E3 > E4 |  |  |  |  |  |  |
| Open Field: Total Distance Moved (cm) ^4^ | F | *F* (2, 87) = 10.703, *p* < 0.001 ^2^ | E3 > E2 & E4 |  |  |  |  |  |  |
| Open Field: Percent Duration in the Center of the Arena (Day 1) | M | *F* (2, 87) = 6.464, *p* = 0.002 | E3 > E2 & E4 |  |  |  |  |  |  |
| Open Field: Percent Duration in the Center of the Arena (Day 1) | F |  |  |  |  |  |  |  |  |
| Y-Maze: % Spontaneous Alternation | M |  |  |  |  |  |  |  |  |
| Y-Maze: % Spontaneous Alternation | F |  |  |  |  |  |  |  |  |
| Y-Maze: Total Arm Entries | M | *F* (2, 87) = 12.562, *p* < 0.001 | E2 > E3 & E4 | *F* (1, 87) = 4.995, *p* = 0.028 | mAPP > NL-G-F |  |  |  |  |
| Y-Maze: Total Arm Entries | F | *F* (2, 80) = 4.816, *p* = 0.011 | E2 > E4 | *F* (1, 80) = 12.503, *p* = 0.001 | mAPP > NL-G-F |  |  |  |  |
| Fear Conditioning: Average Baseline Motion | M |  |  |  |  |  |  |  |  |
| Fear Conditioning: Average Baseline Motion | F | *F* (2, 80) = 15.680, *p* < 0.001 | E3 > E2 & E4 | *F* (1, 80) = 3.197, *p* = 0.078 | mAPP > NL-G-F | *F* (2, 80) = 7.731, *p* = 0.001 | ns | E3 > E2 & E4 | E2: mAPP > NL-G-F  E3: NL-G-F > mAPP  E4: mAPP > NL-G-F |
| Fear Conditioning: Average Motion During Shocks ^4^ | M | *F* (2, 87) = 3.927, *p* = 0.023 ^1^  *F* (2, 87) = 9.797, *p* < 0.001 ^2^ | E2 < E3 & E4 |  |  |  |  |  |  |
| Fear Conditioning: Average Motion During Shocks ^4^ | F | *F* (2, 80) = 7.132, *p =* 0.001 ^2^ | E2 < E3 & E4 | *F* (1, 80) = 5.988, *p =* 0.017 ^2^ | mAPP > NL-G-F |  |  |  |  |
| Fear Conditioning: % Freezing During Tones ^4^ | M |  |  | *F* (1, 87) = 6.780, *p* = 0.011 ^1^ | Tone1 ^1^: NL-G-F > mAPP  Tone2 ^1^: mAPP > NL-G-F |  |  |  |  |
| Fear Conditioning: % Freezing During Tones ^4^ | F |  |  |  |  |  |  |  |  |
| Fear Conditioning: % Freezing During ISI ^4^ | M | *F* (2, 87) = 4.641, *p* = 0.012 ^1^ | ISI1 ^1^: E2 > E3 > E4  ISI2 ^1^: E4 > E2 > E3 | *F* (1, 87) = 7.079, *p =* 0.009 ^2^ | mAPP > NL-G-F | *F* (2, 87) = 3.734, *p* = 0.028 ^1^;  *F* (2, 87) = 6.172, *p =* 0.003 ^2^ | ISI1 ^1^: E4 > E3 > E2  ISI2 ^1^: E4 > E3 & E2  E4 > E2 & E3 ^2^ | ISI1 ^1^: E2 > E4 > E3  ISI2 ^1^: E2 > E4 > E3  ns ^2^ | E2: ns  E3: mAPP > NL-G-F ^2^  E4: mAPP > NL-G-F ^1 & 2^ |
| Fear Conditioning: % Freezing During ISI ^4^ | F |  |  |  |  | *F* (2, 80) = 6.030, *p* = 0.004 ^1^ | ISI1 ^1^: E2 > E3 > E4  ISI2 ^1^: E4 > E2 > E3 | ns ^1^ | E2: ns  E3: ns  E4 ^1^: ISI1 NL-G-F > mAPP  ISI 2 mAPP > NL-G-F |
| Fear Conditioning: % Contextual Freezing | M |  |  |  |  | *F* (2, 87) = 6.096, *p* = 0.003 | ns | E2 > E3 | E2: ns  E3: mAPP > NL-G-F  E4: ns |
| Fear Conditioning: % Contextual Freezing | F | *F* (2, 80) = 4.274, *p* = 0.017 | E2 > E3 & E4 |  |  |  |  |  |  |
| Fear Conditioning: % Cued Freezing | M | *F* (2, 87) = 5.452, *p* = 0.006 | E2 < E3 & E4 |  |  |  |  |  |  |
| Fear Conditioning: %Cued Freezing | F |  |  |  |  |  |  |  |  |
| Fear Conditioning:: % Freezing During Baseline and Tone in Cued Test ^4^ | M | *F* (2, 87) = 5.016, *p* = 0.009 ^1^  *F* (2, 87) = 15.234, *p =* 0.007 ^2^ | E4 > E2 |  |  |  |  |  |  |
| Fear Conditioning:: % Freezing During Baseline and Tone in Cued Test ^4^ | F |  |  |  |  |  |  |  |  |

^1^ within subjects effect; ^2^ between subjects effect. ^3^ Trends that did not reach significance are indicated in red. ^4^ Repeated measures ANOVA. ^5^ Tukey’s Post Hoc, *p* < 0.05.

**Suppl. Table 1B.** Statistical analyses of behavioral performance of mice tested at the 18-month time point ^1,2,3^.

| *Behavioral Measure* | *Sex* | *Effect of APOE* | *Direction of APOE effect ^5^* | *Effect of APP (NL-F)* | *Direction of APP effect* | *APP x APOE interaction* | *Direction of effect in mAPP* | *Direction of effect in APP NL-F* | *Direction of effect in each APOE genotype* |
| --- | --- | --- | --- | --- | --- | --- | --- | --- | --- |
| Average Body Weight | M | *F* (2, 70) = 4.749, *p* = 0.012 | E3 > E4 | *F* (1, 70) = 4.028, *p* = 0.049 | NL-F > mAPP | *F* (2, 70) = 5.446, *p* = 0.006 | E3 < E2 | E2 & E3 > E4 | E2: NL-F > mAPP  E3: ns  E4: ns |
| Average Body Weight | F | *F* (2, 69) = 6.556, *p* = 0.002 | E4 < E2 & E3 | *F* (1, 69) = 7.375, *p* = 0.008 | NL-F > mAPP |  |  |  |  |
| Average Nest Score | M | *F* (2, 70) = 14.667, *p* < 0.001 | E2 < E3 & E4 |  |  |  |  |  |  |
| Average Nest Score | F | *F* (2, 69) = 2.926, *p* = 0.060 | E2 < E3 |  |  |  |  |  |  |
| Change in Nest Score | M |  |  | *F* (1, 70) = 3.167, *p* = 0.079 | NL-F > mAPP |  |  |  |  |
| Change in Nest Score | F |  |  |  |  |  |  |  |  |
| Activity in the Dark Cycle | M |  |  |  |  |  |  |  |  |
| Activity in the Dark Cycle | F | *F* (2, 49) = 4.345, *p* = 0.018 | E3 > E2 & E4 |  |  |  |  |  |  |
| Activity in the Light Cycle | M |  |  |  |  |  |  |  |  |
| Activity in the Light Cycle | F |  |  | *F* (2, 49) = 7.041, *p* = 0.011 | NL-F > mAPP |  |  |  |  |
| Activity Ratio Dark/Light | M |  |  | *F* (1, 52) = 3.359, *p* = 0.073 | mAPP > NL-F |  |  |  |  |
| Activity Ratio Dark/Light | F |  |  |  |  |  |  |  |  |
| Elevated Zero Maze: % Time in the Open Areas | M | *F* (2, 70) = 8.395 *p* = 0.001 | E2 < E3 & E4 | *F* (1, 70) = 5.748 *p* = 0.019 | NL-F > mAPP | *F* (2, 70) = 9.170 *p* < 0.001 | ns | E3 > E4 | E2: NL-F > mAPP  E3: NL-F > mAPP  E4: ns |
| Elevated Zero Maze: % Time in the Open Areas | F | *F* (2, 69) = 2.882 *p* = 0.063 | E3 > E4 |  |  |  |  |  |  |
| Elevated Zero Maze: Entries in Open Areas | M |  |  |  |  |  |  |  |  |
| Elevated Zero Maze: Entries in Open Areas | F | *F* (2, 69) = 13.282 *p* < 0.001 | E4 < E2 & E3 |  |  |  |  |  |  |
| Elevated Zero Maze: Total Distance Moved (cm) | M | *F* (2, 71) = 4.944 *p* = 0.01 | E2 > E3 & E4 |  |  |  |  |  |  |
| Elevated Zero Maze: Total Distance Moved (cm) | F |  |  |  |  |  |  |  |  |
| Wire Hang: Fall Score | M |  |  | *F* (1, 70) = 6.155 *p* = 0.016 | mAPP > NL-F | *F* (2, 70) = 5.228 *p* = 0.008 | E3 < E2 & E4 | ns | E2: mAPP > NL-F  E3: ns  E4: mAPP > NL-F |
| Wire Hang: Fall Score | F |  |  |  |  |  |  |  |  |
| Wire Hang: Reach Score | M | *F* (2, 70) = 5.141 *p* = 0.008 | E3 > E4 |  |  |  |  |  |  |
| Wire Hang: Reach Score | F | *F* (2, 69) = 2.971 *p* = 0.058 | ns |  |  |  |  |  |  |
| Open Field: Total Distance Moved (cm) ^4^ | M |  |  |  |  | *F* (3.196, 111.866) = 2.708 *p* = 0.045 ^1^ | ^1^D1: E2 > E3 > E4  ^1^D2: E2 & E4 > E3  ^1^D3: E2 > E3 > E4 | ns | E2: ^1^D1 mAPP > NL-F, ^1^D2 NL-F > mAPP, ^1^D3 NL-F > mAPP.  E3: ns  E4: ns |
| Open Field: Total Distance Moved (cm) ^4^ | F | *F* (2, 69) = 3.050 *p* = 0.054 ^2^ | ns | *F* (1, 69) = 7.850 *p* = 0.007 ^2^ | NL-F > mAPP |  |  |  |  |
| Open Field: Percent Duration in the Center of the Arena (Day 1) | M |  |  |  |  |  |  |  |  |
| Open Field: Percent Duration in the Center of the Arena (Day 1) | F | *F* (2, 69) = 8.930 *p* < 0.001 | E3 > E2 & E4 |  |  | *F* (2, 69) = 2.851 *p* = 0.065 ^2^ | ns | E3 > E4 | E2: ns  E3: NL-F > mAPP  E4: ns |
| Y-Maze: % Spontaneous Alternation | M |  |  |  |  |  |  |  |  |
| Y-Maze: % Spontaneous Alternation | F |  |  |  |  | *F* (2, 69) = 3.797 *p* = 0.027 | E4 > E3 | ns | E2: ns  E3: NL-F > mAPP  E4: ns |
| Y-Maze: Total Arm Entries | M | *F* (2, 68) = 9.38  *p* < 0.001 | E2 > E3 & E4 |  |  |  |  |  |  |
| Y-Maze: Total Arm Entries | F | *F* (2, 69) = 3.334  *p* = 0.041 | E2 > E4 |  |  |  |  |  |  |
| Fear Conditioning: Average Baseline Motion | M |  |  |  |  |  |  |  |  |
| Fear Conditioning: Average Baseline Motion | F |  |  | *F* (1, 67) = 3.708  *p* = 0.058 | mAPP > NL-F |  |  |  |  |
| Fear Conditioning: Average Motion During Shocks ^4^ | M | *F* (2, 66) = 4.096  *p* = 0.021 ^1^  *F* (2, 66) = 5.249  *p* = 0.008 ^2^ | E3 > E2^2^ | *F* (1, 66) = 12.553  *p* = 0.001 ^1^  *F* (1, 66) = 11.672  *p* = 0.001 ^2^ | mAPP > NL-F^2^ | *F* (2, 66) = 7.761  *p* = 0.001 ^1^ | ns^1^ | Shock1: E3 > E2 & E4 ^1^  Shock2: E4 > E3 > E2 ^1^ | E2: Shock1 NL-F > mAPP ^1^, Shock2 mAPP > NL-F ^1^  E3: Shock1 & Shock2 mAPP > NL-F ^1^  E4: ns^1^ |
| Fear Conditioning: Average Motion During Shocks ^4^ | F | *F* (2, 67) = 5.068  *p* = 0.009 ^1^ | E3 > E4^2^ | *F* (1, 67) = 24.471  *p* < 0.001 ^1^  *F* (1, 67) = 8.126  *p* = 0.006 ^2^ | mAPP > NL-F^2^ | *F* (2, 67) = 4.552  *p* = 0.014 ^1^  *F* (2, 67) = 7.387  *p* = 0.001 ^2^ | ns ^1 & 2^ | Shock1: E3 > E2 > E4 ^1^  Shock2: E3 > E2 & E4 ^1^  E3 > E2 & E4 ^2^ | E2: Shock1 NL-F > mAPP ^1 & 2^, Shock2 mAPP > NL-F ^1 & 2^  E3: Shock1 NL-F > mAPP ^1^, Shock2 mAPP > NL-F ^1^, ns^2^  E4: ns^1^, mAPP > NL-F^2^ |
| Fear Conditioning: % Freezing During Tones ^4^ | M | *F* (2, 66) = 3.319  *p* = 0.042 ^1^  *F* (2, 66) = 2.809  *p* = 0.067 ^2^ | Tone1: E4 > E2 > E3 ^1^  Tone2: E4 > E3 > E2 ^1^  ns^2^ |  |  |  |  |  |  |
| Fear Conditioning: % Freezing During Tones ^4^ | F | *F* (2, 67) = 6.631  *p* = 0.002 ^2^ | E4 > E2 & E3 ^2^ | *F* (1, 67) = 3.326  *p* = 0.073 ^2^ | NL-F > mAPP | *F* (2, 67) = 7.663  *p* = 0.001 ^2^ | ns ^2^ | E4 > E2 & E3 ^2^ | E2: ns ^2^  E3: ns ^2^  E4: NL-F > mAPP |
| Fear Conditioning: % Freezing During ISI ^4^ | M | *F* (2, 66) = 2.772  *p* = 0.070 ^1^  *F* (2, 66) = 12.510  *p* < 0.001 ^2^ | E4 > E2 & E3 ^2^ | *F* (1, 66) = 7.398  *p* = 0.008 ^1^ | ISI1: NL-F > mAPP ^1^  ISI2: mAPP > NL-F ^1^ | *F* (2, 66) = 2.774  *p* = 0.070 ^1^  *F* (2, 66) = 3.605  *p* = 0.033 ^2^ | E4 > E2 ^2^ | E4 > E2 & E3 ^2^ | E2: ns ^2^  E3: mAPP > NL-F  E4: ns ^2^ |
| Fear Conditioning: % Freezing During ISI ^4^ | F | *F* (2, 67) = 9.312  *p* < 0.001 ^2^ | E4 > E2 & E3 ^2^ | *F* (1, 67) = 10.015  *p* = 0.002 ^1^ | ISI1: NL-F > mAPP ^1^  ISI2: mAPP = NL-F ^1^ | *F* (2, 67) = 5.961  *p* = 0.004 ^2^ | ns ^2^ | E4 > E2 & E3 ^2^ | E2: ns ^2^  E3: ns ^2^  E4: NL-F > mAPP |
| Fear Conditioning: % Contextual Freezing | M |  |  | *F* (1, 66) = 3.907  *p* = 0.052 | NL-F > mAPP |  |  |  |  |
| Fear Conditioning: % Contextual Freezing | F | *F* (2, 67) = 7.417  *p* = 0.001 | E4 > E2 & E3 |  |  |  |  |  |  |
| Fear Conditioning: % Cued Freezing | M | *F* (2, 66) = 4.543  *p* = 0.014 | E4 & E3 > E2 | *F* (1, 66) = 3.263  *p* = 0.075 | mAPP > NL-F |  |  |  |  |
| Fear Conditioning: %Cued Freezing | F | *F* (2, 67) = 2.904  *p* = 0.062 | ns | *F* (1, 67) = 10.671  *p* = 0.002 | mAPP > NL-F |  |  |  |  |
| Fear Conditioning:: % Freezing During Baseline and Tone in Cued Test ^4^ | M | *F* (2, 66) = 3.375  *p* = 0.040 ^1^  *F* (2, 66) = 4.409  *p* = 0.016 ^2^ | Baseline: E4 > E2 & E3 ^1^  Tone: E3 & E4 > E2 ^1^  E4 & E3 > E2^2^ |  |  |  |  |  |  |
| Fear Conditioning:: % Freezing During Baseline and Tone in Cued Test ^4^ | F | *F* (2, 67) = .0125  *p* = 0.050 ^1^  *F* (2, 67) = 3.813  *p* = 0.027 ^2^ | E4 > E2 ^2^ | *F* (1, 67) = 13.485  *p* < 0.001 ^1^  *F* (2, 67) = 6.863  *p* = 0.011 ^2^ | Baseline: mAPP = NL-F ^1 & 2^  Tone: mAPP > NL-F ^1 & 2^ |  |  |  |  |

^1^ within subjects effect; ^2^ between subjects effect. ^3^ Trends that did not reach significance are indicated in red. ^4^ Repeated measures ANOVA. ^5^ Tukey’s Post Hoc, *p* < 0.05.

**Suppl. Table 1C.** Statistical analyses of behavioral performance of NL-G-F, NL-F, and WT mice tested at the 18-month time point ^1,2,3^.

| *Behavioral Measure* | *Sex* | *Effect of APP only (WT, NL-F, NL-G-F)* | *Direction of APP only effect^5^* |
| --- | --- | --- | --- |
| Average Body Weight | M | *F* (2, 27) = 7.171  *p* = 0.003 | NL-G-F > NL-F |
| Activity in the Dark Period | M | *F* (2, 16) = 3.423  *p* = 0.058 | NL-G-F > WT |
| Activity in the Light Period | F | *F* (2, 21) = 3.529  *p* = 0.048 | NL-F > WT  NL-G-F > WT |
| Elevated Zero Maze: Entries in Open Areas | M | *F* (2, 27) = 5.545  *p* = 0.010 | NL-F > WT  NL-G-F > WT |
| Elevated Zero Maze: Entries in Open Areas | F | *F* (2, 31) = 10.670  *p <*  0.001 | NL-G-F > WT  NL-G-F > NL-F |
| Wire Hang: Fall Score | M | *F* (2, 27) = 15.116  *p <* 0.001 | WT > NL-G-F  NL-F > NL-G-F |
| Wire Hang: Fall Score | F | *F* (2, 29) = 14.831  *p <* 0.001 | WT > NL-F & NL-G-F  NL-F > NL-G-F |
| Wire Hang: Reach Score | M | *F* (2, 27) = 9.260  *p =* 0.001 | NL-F > NL-G-F  NL-F > WT |
| Open Field: Percent Duration in the Center of the Arena (Day 1) | M | *F* (2, 27) = 7.456  *p =* 0.003 | WT < NL-F & NL-G-F |
| Fear Conditioning: Average Baseline Motion | M | *F* (2, 29) = 6.125  *p =* 0.006 | NL-F > WT & NL-G-F |
| Fear Conditioning: % Contextual Freezing | F | *F* (2, 28) = 4.941  *p =* 0.015 | WT > NL-G-F  NL-F > NL-G-F |
| Fear Conditioning: % Cued Freezing | M | *F* (2, 27) = 13.514  *p <* 0.001 | WT > NL-G-F  NL-F > NL-G-F |
| Fear Conditioning: %Cued Freezing | F | *F* (2, 28) = 4.396  *p =* 0.022 | NL-F > NL-G-F |

^1^ within subjects effect; ^2^ between subjects effect. ^3^ Trends that did not reach significance are indicated in red. ^4^ Repeated measures ANOVA. ^5^ Tukey’s Post Hoc, *p* < 0.05.

**Suppl. Table 1D.** Behavioral performance measures that show APP x APOE interactions at the 6- and 18-month time points ^1,2,3,4^.

| *Behavioral Measure* | *Sex* | *Direction of effect in mAPP* | *Direction of effect in APP NL-G-F* | *Direction of effect in each APOE genotype* |
| --- | --- | --- | --- | --- |
| ***6 months*** |  |  |  |  |
| Average Body Weight | F | E2 > E3 & E4 | E2 > E3 & E4 | E2: mAPP > NL-G-F  E3: mAPP > NL-G-F  E4: NL-G-F > mAPP |
| Average Nest Score | M | E2 < E3 & E4 | ns | E2: NL-G-F > mAPP  E3: ns  E4: ns |
| Average Nest Score | F | E2 < E3 & E4 | ns | E2: NL-G-F > mAPP  E3: ns  E4: ns |
| Change in Nest Score | M | E4 > E2 | E3 < E2 & E4 | E2: NL-G-F > mAPP  E3: ns  E4: ns |
| Activity in the Light Period | F | E2 < E3 & E4 | ns | E2: mAPP < NL-G-F  E3: ns  E4: ns |
| Elevated Zero Maze: % Time in the Open Areas | F | ns | ns | E2: NL-G-F > mAPP  E3: ns  E4: ns |
| Elevated Zero Maze: Entries in Open Areas | M | ns | E3 > E2 & E4 | E2: ns  E3: NL-G-F > mAPP  E4: ns |
| Elevated Zero Maze: Total Distance Moved (cm) | F | ns | E3 < E2 | E2: ns  E3: NL-G-F > mAPP  E4: ns |
| Wire Hang: Reach Score | F | E3 > E4 | ns | E2: NL-G-F > mAPP  E3: ns  E4: NL-G-F > mAPP |
| Fear Conditioning: Average Baseline Motion | F | ns | E3 > E2 & E4 | E2: mAPP > NL-G-F  E3: NL-G-F > mAPP  E4: mAPP > NL-G-F |
| Fear Conditioning: % Freezing During ISI ^4^ | M | ISI1 ^1^: E4 > E3 > E2  ISI2 ^1^: E4 > E3 & E2  E4 > E2 & E3 ^2^ | ISI1 ^1^: E2 > E4 > E3  ISI2 ^1^: E2 > E4 > E3  ns ^2^ | E2: ns  E3: mAPP > NL-G-F ^2^  E4: mAPP > NL-G-F ^1 & 2^ |
| Fear Conditioning: % Freezing During ISI ^4^ | F | ISI1 ^1^: E2 > E3 > E4  ISI2 ^1^: E4 > E2 > E3 | ns ^1^ | E2: ns  E3: ns  E4 ^1^: ISI1 NL-G-F > mAPP  ISI 2 mAPP > NL-G-F |
| Fear Conditioning: % Contextual Freezing | M | ns | E2 > E3 | E2: ns  E3: mAPP > NL-G-F  E4: ns |
| ***18 months*** |  |  |  |  |
| Average Body Weight | M | E3 < E2 | E2 & E3 > E4 | E2: NL-F > mAPP  E3: ns  E4: ns |
| Elevated Zero Maze: % Time in the Open Areas | M | ns | E3 > E4 | E2: NL-F > mAPP  E3: NL-F > mAPP  E4: ns |
| Wire Hang: Fall Score | M | E3 < E2 & E4 | ns | E2: mAPP > NL-F  E3: ns  E4: mAPP > NL-F |
| Open Field: Total Distance Moved (cm) ^4^ | M | ^1^D1: E2 > E3 > E4  ^1^D2: E2 & E4 > E3  ^1^D3: E2 > E3 > E4 | ns | E2: ^1^D1 mAPP > NL-F, ^1^D2 NL-F > mAPP, ^1^D3 NL-F > mAPP.  E3: ns  E4: ns |
| Open Field: Percent Duration in the Center of the Arena (Day 1) | F | ns | E3 > E4 | E2: ns  E3: NL-F > mAPP  E4: ns |
| Y-Maze: % Spontaneous Alternation | F | E4 > E3 | ns | E2: ns  E3: NL-F > mAPP  E4: ns |
| Fear Conditioning: Average Motion During Shocks ^4^ | M | ns^1^ | Shock1: E3 > E2 & E4 ^1^  Shock2: E4 > E3 > E2 ^1^ | E2: Shock1 NL-F > mAPP ^1^, Shock2 mAPP > NL-F ^1^  E3: Shock1 & Shock2 mAPP > NL-F ^1^  E4: ns^1^ |
| Fear Conditioning: Average Motion During Shocks ^4^ | F | ns ^1 & 2^ | Shock1: E3 > E2 > E4 ^1^  Shock2: E3 > E2 & E4 ^1^  E3 > E2 & E4 ^2^ | E2: Shock1 NL-F > mAPP ^1 & 2^, Shock2 mAPP > NL-F ^1 & 2^  E3: Shock1 NL-F > mAPP ^1^, Shock2 mAPP > NL-F ^1^, ns^2^  E4: ns^1^, mAPP > NL-F^2^ |
| Fear Conditioning: % Freezing During Tones ^4^ | F | ns ^2^ | E4 > E2 & E3 ^2^ | E2: ns ^2^  E3: ns ^2^  E4: NL-F > mAPP |
| Fear Conditioning: % Freezing During ISI ^4^ | M | E4 > E2 ^2^ | E4 > E2 & E3 ^2^ | E2: ns ^2^  E3: mAPP > NL-F  E4: ns ^2^ |
| Fear Conditioning: % Freezing During ISI ^4^ | F | ns ^2^ | E4 > E2 & E3 ^2^ | E2: ns ^2^  E3: ns ^2^  E4: NL-F > mAPP |

^1^ within subjects effect; ^2^ between subjects effect. ^3^ Trends that did not reach significance are indicated in red. ^4^
