# Supplementary figures and images for: Apolipoprotein E Isoform-Dependent Effects on Human Amyloid Precursor Protein/Aβ-Induced Behavioral Alterations and Cognitive Impairments and Insoluble Cortical Aβ42 Levels
Source: Front Aging Neurosci. 2022 Mar 1;14:767558. doi: 10.3389/fnagi.2022.767558 (PMC8922030; doi:10.3389/fnagi.2022.767558)

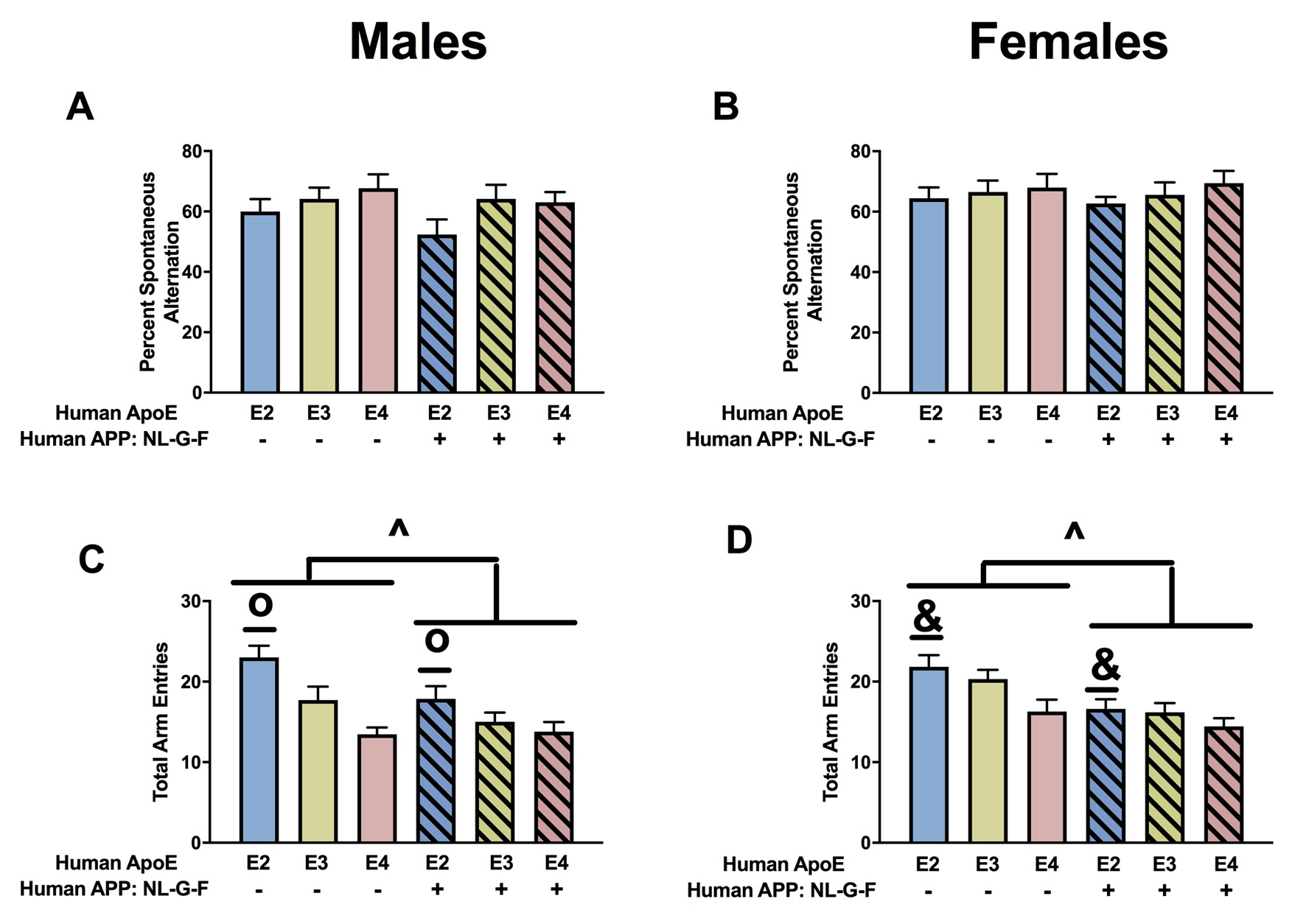

Supplement: Supplementary Figure 1 — Performance of males (A,C) and females (B,D) in the Y maze at the 6-month time point. (A) Spontaneous alternation of males at the 6-month time point. (B) Spontaneous alternation of females at the 6-month time point. (C) There was an effect of APOE and APP on activity levels of males in the elevated zero maze. E2 males were more active in the elevated zero maze than E3 and E4 males. op < 0.05. In addition, NL-G-F mice were less active. ^p < 0.05. (D) There also was an effect of APOE and APP on activity levels of females in the elevated zero maze. E2 females were more active in the elevated zero maze than E4 females. &p < 0.05. In addition, NL-G-F mice were less active. ^p < 0.05. [file Image_1.JPEG]

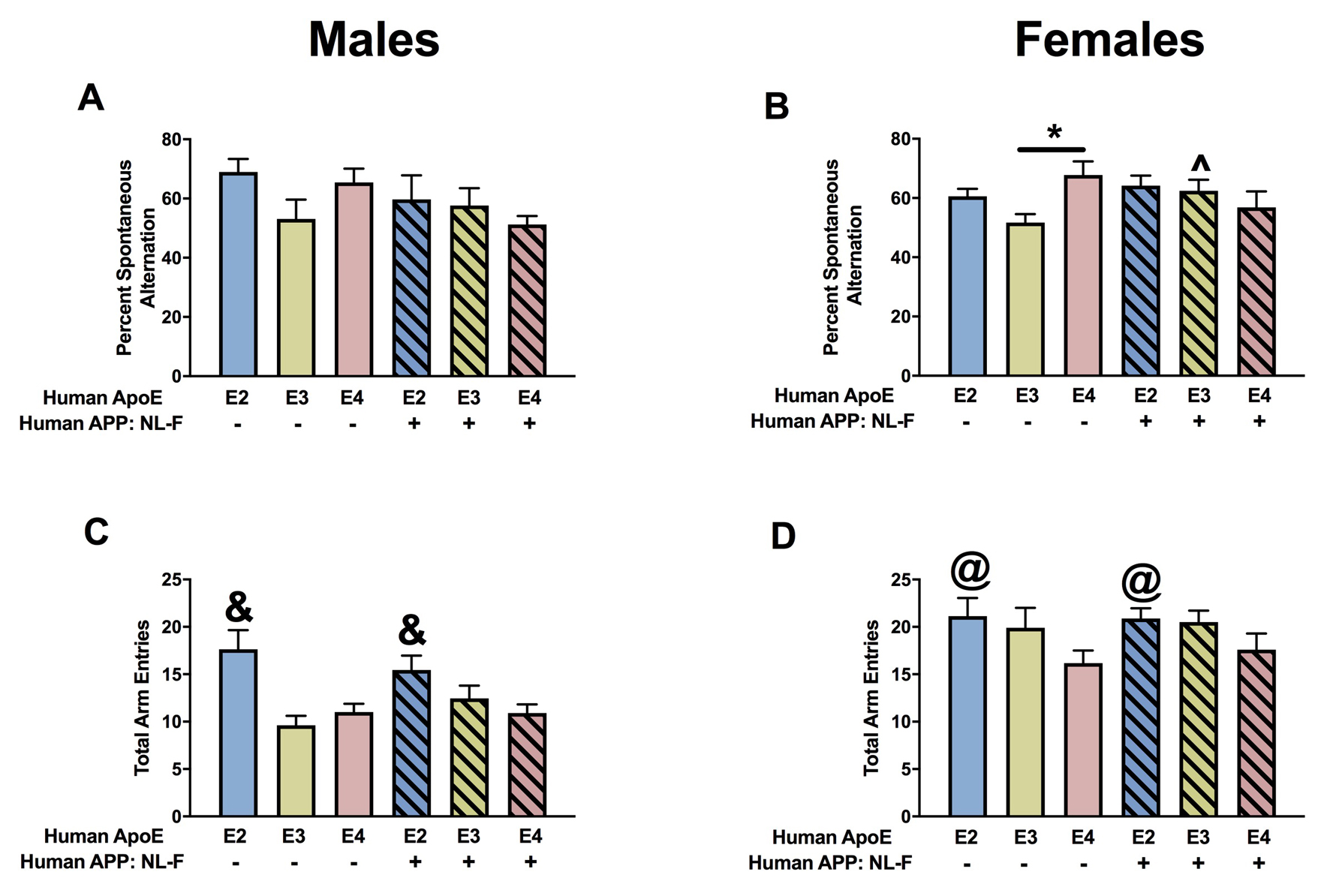

Supplement: Supplementary Figure 2 — Performance of males (A,C) and females (B,D) in the Y maze at the 18-month time point. (A) Spontaneous alternation of males at the 18-month time point. (B) There was an APP × APOE interaction for spontaneous alternation of females at the 18-month time point. E4 females showed more spontaneous alternation than E3 females. *p < 0.05. In addition, NL-F/E3 females showed more spontaneous alternation than E3 females. ^p < 0.05. (C) There was an effect of APOE on activity levels of males in the Y maze. Activity levels were higher in E2 than E3 and E4 males. &p < 0.05. (D) There was an effect of APOE on activity levels of females in the Y maze. Activity levels were higher in E2 than E4 females. @p < 0.05. [file Image_2.JPEG]

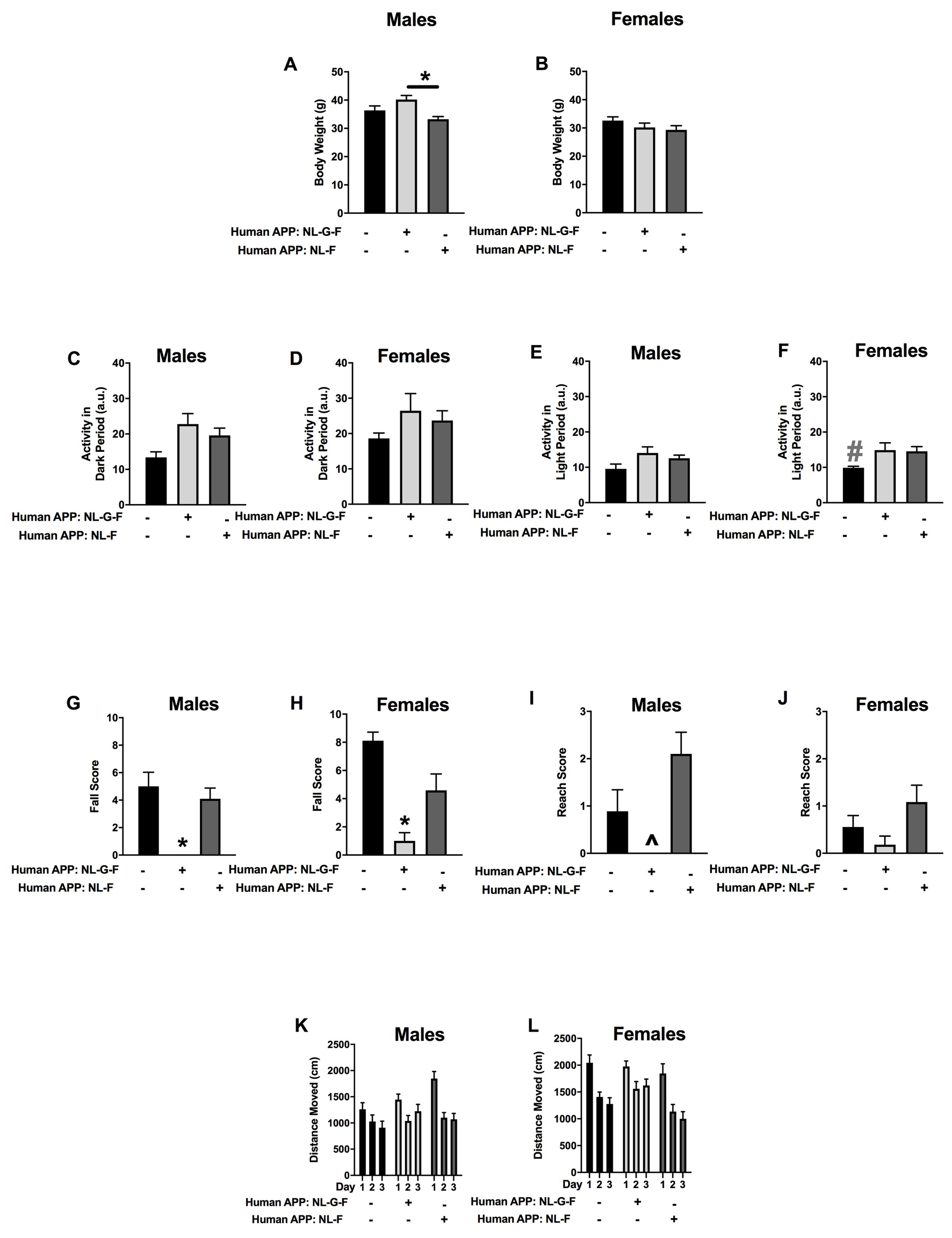

Supplement: Supplementary Figure 3 — Body weights, home cage activity, performance in the wire hang, and open field of male (A,C,E,G,I,K) and female (B,D,F,H,J,L) NL-G-F, NL-F, and WT mice at the 18-month time point. (A) There was an effect of APP on body weights of males. NL-G-F males were heavier than NL-F males. *p < 0.05. (B) Body weights of NL-G-F, NL-F, and WT female mice. (C) There was a trend toward an effect of APP on activity of males during the dark period. There was a trend toward higher activity levels in NL-G-F than WT males. (D) Activity levels of NL-G-F, NL-F, and WT females during the dark period. (E) Activity levels of NL-G-F, NL-F, and WT males during the light period. (F) There was an effect of APP on activity levels of females during the light period. There was a trend of NL-F and NL-G-F females being more active during the light period than WT females. (G) There was an effect of APP on fall scores of males in the wire hang test. NL-G-F males had lower fall scores than NL-F and WT males. *p < 0.05. (H) There was an effect of APP on fall scores of females in the wire hang test. NL-G-F females had lower fall scores than NL-F and WT females. (I) There was an effect of APP on reach scores of males in the wire hang test. NL-G-F males had lower reach scores than NL-F males. ^p < 0.05. (J) Reach scores of NL-G-F, NL-F, and WT females. (K) Activity levels of NL-G-F, NL-F, and WT males in the open field. (L) Activity levels of NL-G-F, NL-F, and WT females in the open field. [file Image_3.JPEG]

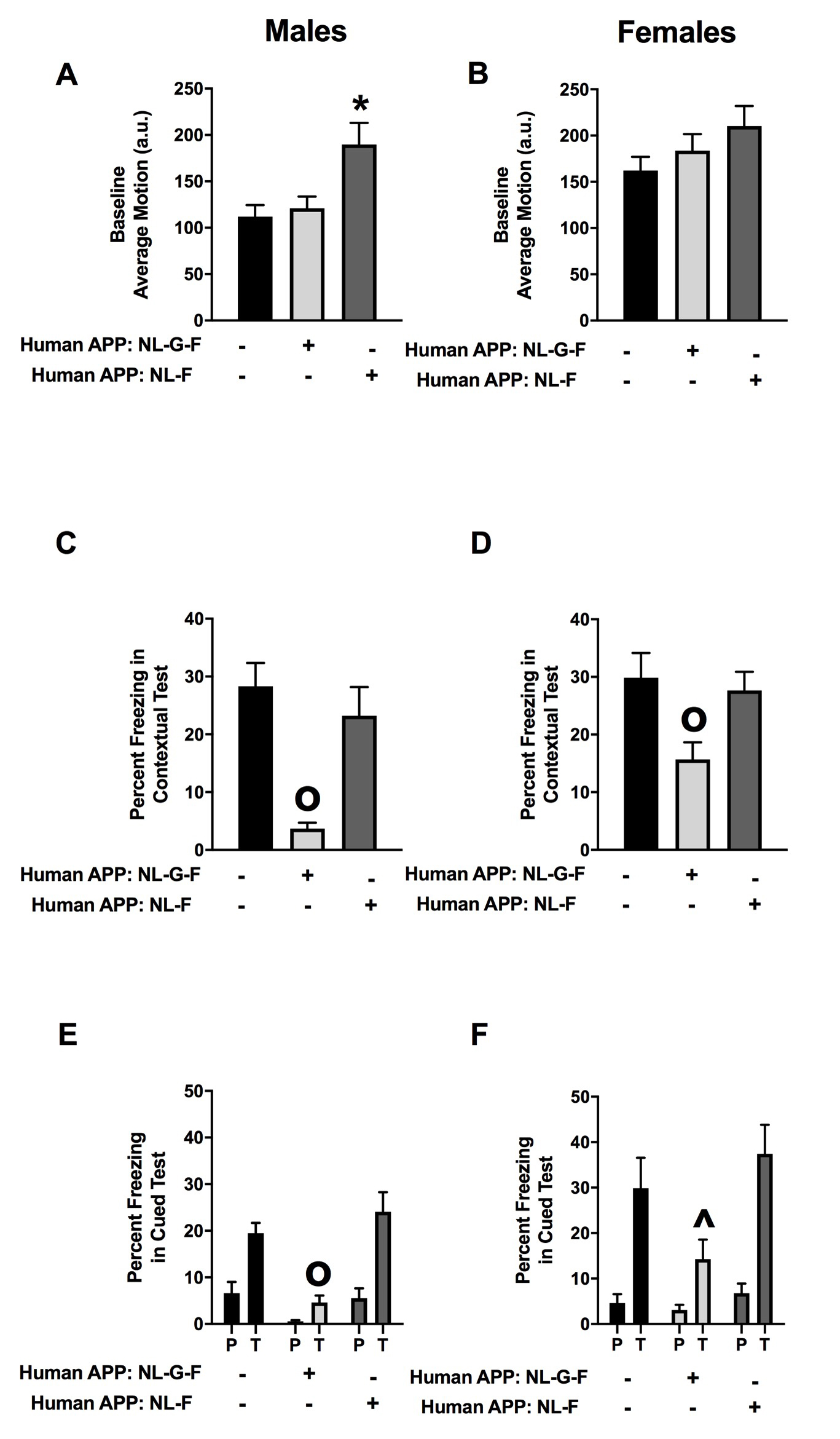

Supplement: Supplementary Figure 4 — Performance of NL-G-F, NL-F, and WT males (A,C,E) and females (B,D,F) at the 18-month time point. (A) There was an effect of APP on baseline motion of the males. NL-F males moved more during the baseline period than NL-G-F and WT males. *p < 0.05. (B) Activity levels of NL-G-F, NL-F, and WT females during the baseline period. (C) There was an effect of APP on contextual fear memory of males. NL-G-F males froze less than NL-F and WT males during the contextual fear memory test. op < 0.05. (D) There was an effect of APP on contextual fear memory of females. NL-G-F females also froze less than NL-F and WT females during the contextual fear memory test. op < 0.05. (E) There was an effect of APP on cued fear memory of males. NL-G-F males froze less during the tone than NL-F and WT males. op < 0.05. (F) There was an effect of APP on cued fear memory of females. NL-G-F females froze less during the tone than NL-F females. ^p < 0.05. [file Image_4.JPEG]
